# Model-based clustering with certainty estimation: implication for clade assignment of influenza viruses

**DOI:** 10.1186/s12859-016-1147-x

**Published:** 2016-07-21

**Authors:** Shunpu Zhang, Zhong Li, Kevin Beland, Guoqing Lu

**Affiliations:** Department of Statistics, University of Central Florida, Orlando, FL, 32816 USA; College of Science, Zhejiang Sci-Tech University, Hangzhou, 310018 China; Department of Biology, University of Nebraska at Omaha, Omaha, NE 68182 USA

**Keywords:** Model-based clustering, Multidimensional scaling, Bootstrap, Certainty, Influenza A hemagglutinin (HA)

## Abstract

**Background:**

Clustering is a common technique used by molecular biologists to group homologous sequences and study evolution. There remain issues such as how to cluster molecular sequences accurately and in particular how to evaluate the certainty of clustering results.

**Results:**

We presented a model-based clustering method to analyze molecular sequences, described a subset bootstrap scheme to evaluate a certainty of the clusters, and showed an intuitive way using 3D visualization to examine clusters. We applied the above approach to analyze influenza viral hemagglutinin (HA) sequences. Nine clusters were estimated for high pathogenic H5N1 avian influenza, which agree with previous findings. The certainty for a given sequence that can be correctly assigned to a cluster was all 1.0 whereas the certainty for a given cluster was also very high (0.92–1.0), with an overall clustering certainty of 0.95. For influenza A H7 viruses, ten HA clusters were estimated and the vast majority of sequences could be assigned to a cluster with a certainty of more than 0.99. The certainties for clusters, however, varied from 0.40 to 0.98; such certainty variation is likely attributed to the heterogeneity of sequence data in different clusters. In both cases, the certainty values estimated using the subset bootstrap method are all higher than those calculated based upon the standard bootstrap method, suggesting our bootstrap scheme is applicable for the estimation of clustering certainty.

**Conclusions:**

We formulated a clustering analysis approach with the estimation of certainties and 3D visualization of sequence data. We analysed 2 sets of influenza A HA sequences and the results indicate our approach was applicable for clustering analysis of influenza viral sequences.

**Electronic supplementary material:**

The online version of this article (doi:10.1186/s12859-016-1147-x) contains supplementary material, which is available to authorized users.

## Background

Clustering is a common technique used in biology, which partitions molecular sequence data or gene expression data into groups such that the data points are highly similar within group but different between/among groups [1, 2]. In general, clustering methods are divided into 2 categories: the non-model-based (distance/similarity-based) approaches and the model-based approaches [3, 4]. The widely used *k*-means method, as well as its variants, is a non-model based method. Model-based clustering techniques can be traced at least as far back as 1963 [5]. In model-based clustering the data are assumed from a finite mixture of different probability models such as the multivariate normal distributions [6–9]. With the underlying probability models, the number of clusters and the parameters in the probability models are estimated using statistical methods such as the expectation maximization (EM) algorithm. A review of model-based clustering can be found in [10].

The clustering methods can also be divided into partitional clustering and hierarchical clustering [3, 4]. A partitional clustering method divides the data objects into *M* (often specified a priori) groups according to some optimization criteria. The *k*-means algorithm is a classic example of partitional methods [1]. A hierarchical method builds a hierarchical set of nested clusters, with the clustering at the top level containing a single cluster of all data objects, and the clustering at the bottom level containing singleton clusters (i.e., 1 cluster for each data object). The resulting hierarchy shows at each level 2 clusters are merged together with the inter-cluster distance presented, and thus provides a good visualization tool. The Single-Link, Complete-Link and Average-Link Clustering methods are examples of hierarchical clustering.

Influenza virus is an important pathogen not only to humans, but also to many other animals such as birds and pigs [11, 12]. The influenza virus can evolve rapidly to avoid detection and neutralization by the host immune system. Detecting new viral strains is thus an ongoing task for improved influenza surveillance and control. To this end, the WHO (World Health Organization), OIE (World Organization for Animal Health) and FAO (Food and Agriculture Organization of the United Nations) H5N1 Evolution Working Group developed a clade nomenclature system for Eurasian highly pathogenic avian influenza (HPAI) A (H5N1) viruses [11, 13]. Several statistical and bioinformatics issues remain unresolved, including how to cluster the sequences more accurately, how to visualize the clustering results intuitively, and how to evaluate the certainty of the sequences within a cluster and the certainty of a cluster.

In our previous study, 2 dimensional scaling analysis was conducted on 109 HA sequences of well-represented HPAI H5N1 viruses to evaluate the above clade designation system by WHO/OIE/FAO [14]. In this paper, we focused on the model-based clustering approach due to its flexibility in finding meaningful clusters in the data and applied this approach to analyze influenza viral sequences. In addition, we designed a subset bootstrap scheme and applied it to estimate the certainty of a given sequence assigned to a particular cluster as well as the certainty of clustering (the stability of the clusters when being considered together).

## Methods

### Similarity estimation and visualization

In our proposed method, pairwise distances of aligned sequences were computed using the identity matrix. The resulting matrix contains the squared root of pairwise distances. Many existing methods cluster sequences by directly applying clustering algorithms (model or non-model-based) to pairwise distances. We do not recommend this approach since many of such methods (especially the model-based methods) require independence of the data and this requirement is not satisfied due to the dependence of the pairwise distances. Instead, we apply the multidimensional scaling (MDS) methods to the distance matrix to explore the similarity or dissimilarity features in the data by assigning a location, i.e., the coordinates in a *d*-dimensional space, to each sequence, where *d* is specified a priori. The MDS method can provide the location data that closely preserves the pairwise distances. The MDS is a statistical method often used in data visualization for exploring similarities or dissimilarities of objects in a parsimonious way. Other methods that have similar property as the MDS methods include the principle component analysis, among others. With the location data available in the *d*-dimensional space, the corresponding sequences can be visualized using graphical tools.

### Mixture model analysis

Denote the location of a sequence generated by MDS in the *d*-dimensional space by x = (*x*_1_,…,*x*_*d*_). In the multivariate normal mixture model, the location data obtained from MDS are assumed from a mixture of multivariate normal distributions where each distribution represents a cluster. The multivariate normality assumption is not a very restrictive assumption. It has been demonstrated in the literature that a non-normal component in the data can often be approximated by several normal ones [15, 16]. Assume that there are *s* sequences, and the *s* data points *x*_1_,…,*x*_*s*_ generated using MDS are from a *g*-variate normal distribution *N* (μ_*k*_, ∑_*k*_), where μ_*k*_ is the mean vector and ∑_*k*_ is the covariance matrix, the likelihood function of *x*_1_,…,*x*_*s*_ is

1 $$ L\left({\boldsymbol{\uptheta}}_1,\dots, {\boldsymbol{\uptheta}}_G;{\pi}_1,\dots, {\pi}_G\Big|x\right)={\displaystyle \prod_{i=1}^s{\displaystyle \sum_{k=1}^g{\pi}_k{f}_k\left({\mathbf{x}}_i\Big|{\boldsymbol{\uptheta}}_k\right)}}, $$

where *f*_*k*_ is the *d*-variate normal density of *N* (μ_*k*_, ∑_*k*_), θ_*k*_ = (μ_*k*_, ∑_*k*_) and π_*k*_ (≥0) is the probability that an observation is from *N* (μ_*k*_, ∑_*k*_), the *k*-th cluster, satisfying ${\displaystyle \sum_{k=1}^g{\pi}_k=1}$.

The multivariate mixture model (1) can be fit by using the R-package Mclust [15]. The optimal model is selected by comparing the Bayesian Information Criterion (BIC) value of each model. The BIC is the value of the maximized log-likelihood with a penalty for the number of parameters in the model, and allows comparison of models with different parameterizations and/or different numbers of clusters. As stated in [15], in general the larger the value of the BIC, the stronger the evidence for the model and the number of clusters. Based upon the fitted multivariate normal mixture model, the sequences can be assigned to the clusters according to their highest posterior probabilities.

### Certainty estimation

An important question about a clustering method is the certainty in the clustering results. There are actually 2 related questions: the certainty in the clustering of individual sequences in a cluster and the certainty of clusters.

### The certainty of individual sequences in a cluster

Denote by z_*i*_ = (z_*i*_,…,z_*ig*_) the conditional (or posterior) probability vector of the observed sequence *i*, is classified to the clusters, 1 ≤ *i* ≤ *s*, where *g* is the number of clusters determined. We define the certainty associated with sequence *i* as max (z_*i*_), which is the probability that sequence *i* belongs to the cluster in which it has been classified. To summarize the certainties in the classification of individual sequences, we use the 5 number summary (the minimum, 25 % quantile, the median, the 75 % quantile, the maximum) of {max (z_*i*_), 1 ≤ *i* ≤ *s*} as an overall measure of certainty in the classification of all sequences. Fraley and Raftery [9] used the concept of uncertainty instead of certainty which is simply equal to 1 minus the certainty. For sequence analysis, it is more convenient to use certainty as a measure of stability in clustering instead of uncertainty.

### The certainty of clusters

#### The subset bootstrap method

Bootstrapping is a well-accepted and widely used method based upon random sampling with replacement method to estimate support values for clustering or groupings. It is a method vertically drawing samples with replacement, and hence can mimic molecular evolution events such as substitution, deletion or insertion. The general practice of bootstrapping molecular sequences is to resample the whole set of sequences [17, 18]. More specifically, the aligned sequences are formed as a matrix with each sequence as a row of the matrix. The bootstrap method constructs a bootstrap data by re-sampling all columns of the original sequence data matrix with replacement. The standard bootstrap method assumes the independence of the columns of the aligned sequences [18].

The assumption of independence among the nucleotide bases of a DNA sequence is obviously questionable. Bootstrap methods for dependent data are an active research area. Some of the well-known methods are the subsampling method and the block bootstrap method [19], among others. In the subsampling method random subsamples of consecutive observations of length (<*n*, where *n* is the length of the whole sequemce) are taken from the whole sequence. The subsampling method has quite universal applicability. However, a poor rate of convergence has been shown in literature [20]. In the block bootstrap method blocks of consecutive observations are drawn with replacement from a set of blocks. The block bootstrap is a very powerful method for dependent data and has a very broad range of applications. Nevertheless, it is hard to justify its use for re-sampling DNA sequences. In this paper, we argue a more appropriate way to mimic natural evolution is to re-sample only a randomly selected subset of the nucleic acid bases of the sequences while keeping the remaining of the sequences fixed.

We propose a subset bootstrap method, where the practitioner first decides the proportion of the sequence being sampled, and bootstrapping is then conducted by randomly choosing this proportion of the nucleic acid bases of the DNA sequences as the subset for re-sampling, while keeping the remaining sequence unchanged. Specific to our sequence data, we first randomly select a subset of columns from the aligned sequences according to the pre-determined proportion. Then, the standard bootstrap procedure is applied to the positions of the selected columns in the subset to obtain a bootstrap sample. The obtained new matrix is called a subset bootstrap sample. After a subset bootstrap sample of sequences is available, the finite mixture model is fitted to the subset bootstrap sample, and clustering is conducted based on the newly fitted finite mixture model.

A reasonable way to choose an appropriate proportion of subsampling in the subset bootstrap method is to use the average substitution rate among observed sequences under study. More specifically, we calculate the substitution rate from each pair of observed sequences, and then use the average rate as the proportion of sub-sampling in the subset bootstrapping method. A more sophisticated way of determining the proportion of subsampling is to randomly select a value from the rates of changes calculated from each pair of observed sequences, i.e., to apply the bootstrap method to the pairwise rates of changes. However, for simplicity we will only use the average rate as the proportion of subsampling in this paper.

#### Evaluation of the certainty of clusters

One difficulty in evaluating the certainty/uncertainty of clusters is how to define the similarity of 2 clusters. Well-known similarity measures include the Jaccard coefficient [21–23], among others. We decided to use the Jaccard coefficient due to its simplicity and other appealing features [24]. The Jacard coefficient is defined as follows:

$$ \gamma \left(C1,C2\right)=\frac{\left|C1\cap C2\right|}{\left|C1\cup C2\right|}, $$

where *C*1, *C*2 are any 2 clusters and |▪| is the number of elements in the set. It can be easily seen that the Jaccard coefficient is 1 when 2 clusters are the same, is zero if 2 clusters are disjoint, and is between 0 and 1, otherwise. Some theoretical justifications for the use of the Jaccard coefficient to compare clusters can be found in [24, 25].

Denote by *C* = {*C*_1_,…,*C*_*i*_…,*C*_*I*_} the clustering obtained from the original data, where *C*_*i*_ is the i^th^ cluster and *I* is the number of clusters. For any given cluster *C*_*i*_, we evaluate its certainty as follows:

Given a pre-determined bootstrapping proportion *p* and let *b* be the index of the bootstrap sample from the subset bootstrap sampling, *b* = 1,…,*B*. For each *b*,

Step 1: Simulate the subset bootstrap sequences using the subset bootstrap method.

Step 2: Apply Mclust to the bootstrapping sample to obtain a new clustering, denoted as ${\overset{\sim }{C}}_b=\left\{{C}_1^{\ast },\dots, {C}_{J_b}^{\ast}\right\},$, where *C*_j_^*^ is the j^th^ clusters and *J*_*b*_ is the number of clusters of the new clustering, which may be different from the number of clusters of the original clustering, it may also be different depending on bootstrap samples.

Step 3: Calculate the maximum Jaccard coefficient between *C*_*i*_ and each cluster *C*_j_^*^ in the new clustering ${\tilde{C}}_b$ and define it as the Jaccard coefficient between *C*_*i*_ and ${\tilde{C}}_b$, i.e.,

$$ J\left({C}_i,{\overset{\sim }{C}}_b\right)={ \max}_{\kern.2em 1\le j\le {J}_b}\left\{J\left({C}_i,{C}_j^{\ast}\right)\right\}, $$

where

$$ i=1,\dots, I;b=1,\dots, B. $$

The certainty of cluster *C*_*i*_ is defined as

2 $$ J\left({C}_i\right)={\displaystyle {\sum}_{b=1}^BJ\left({C}_i,{\tilde{C}}_b\right)/B,} $$

where

$$ i=1,\dots, I. $$

To estimate certainty between two clusterings, the Jaccard coefficient is the proportion of observation pairs (observed sequence pairs) belonging to the same cluster in both clusterings out of all the observation pairs belonging to the same cluster in at least 1 of the clustering. Specifically, let *C*_1_,*C*_2_ be 2 clusterings, and let *n*_11_ be the number of observation pairs belonging to the same cluster in both clusterings; *n*_10_ be the number of observation pairs belonging to the same cluster in the first clustering, but not the second clustering; and *n*_01_ be the number of observation pairs belonging to the same cluster in the second clustering, but not the first clustering. The Jaccard coefficient is defined as

3 $$ J\left({C}_1,{C}_2\right)=\frac{n_{11}}{n_{11}+{n}_{10}+{n}_{01}}. $$

Using the clusters obtained from the mixture model analysis of the original sequences of avian influenza viruses, we use the following algorithm to calculate the overall certainty of the clusters. Given a pre-determined bootstrapping proportion *p* and let *b* be the index of the bootstrap sample, *b* = 1,…,*B*. For each *b*,

First, repeat Steps 1 and 2 from the previous algorithm.

Step 3: Calculate $J\left(C,{\tilde{C}}_b\right)$, *b* = 1,…,*B*, where $C,{\tilde{C}}_b$ are defined the same as before, i.e., they are the original clustering and the clustering obtained from the *b*^*th*^ subset-bootstrap sample, respectively.

With $J\left(C,{\tilde{C}}_b\right)$ being defined as (3), we define

4 $$ J(C)={\displaystyle {\sum}_{b=1}^BJ\left(C,{\tilde{C}}_b\right)/B,} $$

as the overall certainty of clustering *C*.

### Data sets and analysis

Experimental dataset 1, kindly provided by the WHO/OIE/FAO H5N1 Evolution Working Group (RO Donis, personal communication), includes 109 HA sequences of HPAI H5N1 viruses, each with approximately 1,659 nucleotides. These sequences were selected from vaccine strains, reference strains, human isolates, pathogenesis study strains, and geographically diverse isolates in order to establish a unified system to name existing clades of highly pathogenic H5N1 avian influenza A viruses [11, 13].

Experimental dataset 2 includes 1,168 HA sequences of all influenza A viruses with a H7 subtype, each with approximately 1,650 bp, downloaded from fludb (http://www/fludb.org). The sequences were aligned with ClustalW [26] and the alignment was carefully checked and manually edited using BioEdit 7.0 (http://www.mbio.ncsu.edu/bioedit/bioedit.html). The clustering analysis was conducted using the R-package. The aligned sequences were analyzed using dist.alignment function in the R package Seqinr to compute pairwise distances using identity matrix. The multidimensional scaling (MDS) analysis was then conducted using the cmdscale module. We used the function plot3d rgl to display the influenza sequences in 3D space and employed clusteval to calculate the overall cluster certainty.

## Results and discussion

**Fig. 1:**
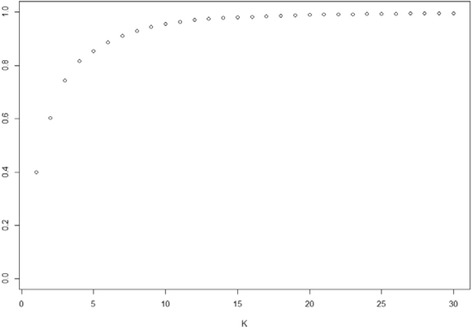
Mardia criterion for selecting d, the number of dimensions for MDS

**Fig. 2:**
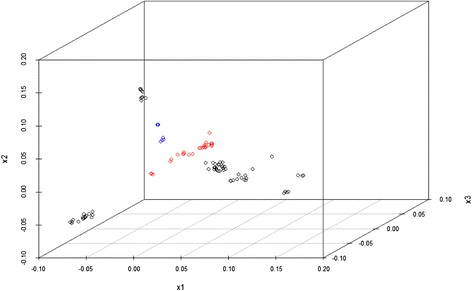
The 3D MDS plot of highly pathogenic avian influenza (HPAI) H5N1 HA sequences

**Fig. 3:**
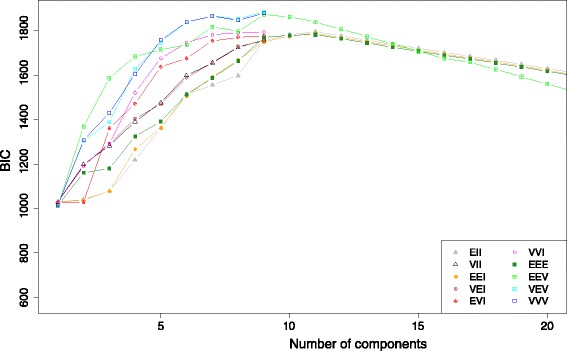
The BIC values corresponding to different numbers of clusters for highly pathogenic avian influenza (HPAI) H5N1 HA sequences

**Fig. 4:**
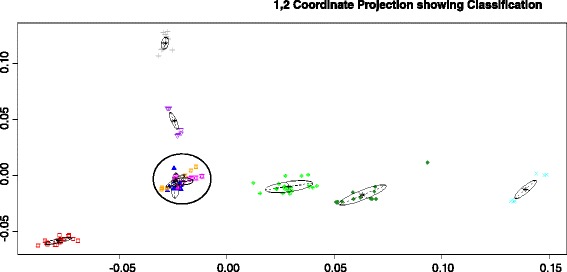
The Mclust results from 3D-MDS location data of highly pathogenic avian influenza (HPAI) H5N1 HA sequences

**Fig. 5:**
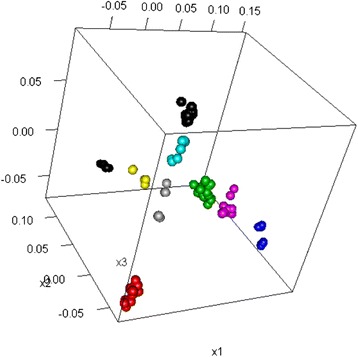
The 3D representation of highly pathogenic avian influenza (HPAI) H5N1 HA sequences

The experimental dataset 1 was studied previous in [13], where a 2D MDS was used to visualize structure of HPAI H5N1 HA sequence data. Further investigation identifies 2 issues: 1) the 2D MDS may not be an optimal way to represent the complexity of the sequence data; 2) there is no estimation of confidence level for individual sequences or specific clusters. To address the first issue, we used the criterion suggested in [27] to select the dimension of MDS (*d*). The Mardia criterion (a parameter used for determining the number of dimensions that considerably differ) shows significant increases for *d* from 1 to 2 and from 2 to 3 (Fig. 1), and after that the increase becomes less obvious. Therefore, we chose *d* = 3. Figure 2 shows the corresponding 3D MDS plot of the H5N1 HA influenza sequences, which obviously provides better separation between clusters (i.e., clades). Figure 3 displays the BIC values for different numbers of clusters. It can be seen clearly that the optimal number of clusters according to BIC values of VEV, VVV, and EEV models is 9, with corresponding BIC values being 1886.6, 1881.2, and 1874.9, respectively. Figure 4 shows the 9 clusters identified by Mclust for the HPAI H5N1 HA sequences. Figure 5 provides a snapshot of the 3D plot, where 9 clusters are clearly depicted.

We compared the clusters obtained from Mclust based on the 3D MDS and those from the clade designation of WHO. There is a general consensus between the clusters obtained in the present study from Mclust and designed previously by WHO (Additional file 1: Table S1). We calculated the certainties of individual sequences in specific clusters using the method described and found that for all 109 sequences the certainties assigned to a specific clade are all equal to 1.0, which indicates that the sequences within cluster are very similar and that the sequences between the clusters are distinct. To summarize the uncertainties in the classification of individual sequences, we obtained the 5 number summary (the minimum, 25 % quantile, the median, the 75 % quantile, the maximum) of {max (z_*i*_), 1 ≤ *i* ≤ *s*} as an overall measure of certainty in the classification of all sequences. Not surprisingly, for this dataset the certainties in the 5 number summary are all 1.0.

In order to conduct the subset bootstrap method to evaluate the certainty of clustering obtained from the mixture model method, we first calculated the average substitution rate (0.075) from the H5N1 HA sequences. Then we used this rate for the subset bootstrap. The certainties of clusters and the clustering are then calculated using (2) and (4), respectively. The clustering *C* = {*C*_1_,…,*C*_9_}, the reference clustering in (2) and (4) for calculating the certainties, was obtained by applying model based method (1) to the original data and is reported in the first column of Additional file 1: Table S1. It can be seen that {*C*_1_,…,*C*_9_} is consistent with the WHO-curated clade information. Each cluster is either a clade, a subclade, a combination of clades, or a combination of subclades of the same clade. We have not observed any case in which 2 influenza sequences belonging to the same clade or subclade were assigned to different clusters.

Table 1 shows that the certainties of given sequences assigned to a specific cluster are high, ranging from 0.92 to 1.0 and the overall certainty of clustering is 0.95 (Table 1). For comparison, we included the results from the standard bootstrap method (i.e., the subset bootstrap method with proportion *p* = 100 %) in Table 1. Apparently, as we expected, the certainty values estimated using the subset bootstrap method are all higher than those estimated using the standard bootstrap method, suggesting our bootstrap scheme is practicable.

**Table 1:** The certainties of clusters and overall clustering for highly pathogenic avian influenza HPAI H5N1 HA sequences

Cluster ID	Cluster									Overall
	1	2	3	4	5	6	7	8	9	
Subset-bootstrap (7.5 %)	0.93	1.00	0.98	0.97	0.92	0.96	1.00	0.99	1.00	0.95
Standard bootstrap method	0.76	0.99	0.72	0.90	0.67	0.66	0.83	0.76	0.97	0.69

**Fig. 6:**
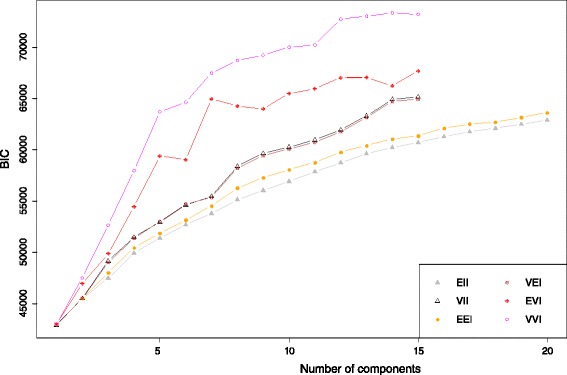
The BIC values corresponding to different numbers of clusters for influenza H7 HA sequences

**Fig. 7:**
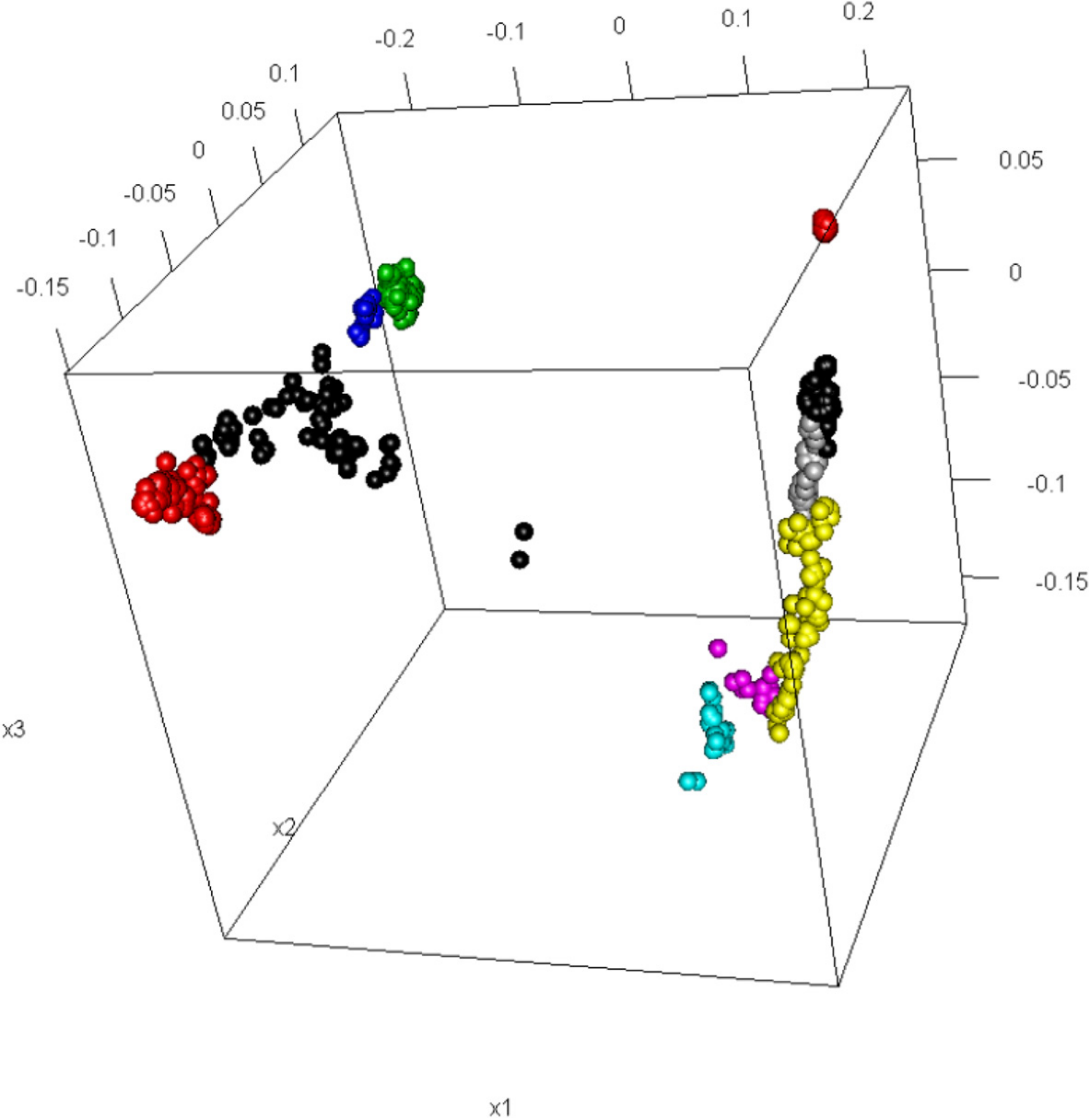
The 3D representation of 10 clusters for influenza H7 HA sequences

It is tempting to fit the Mclust directly to the pairwise distance matrix due to its simplicity. However, Fig. 6 shows that the resulting BIC plot of influenza H7 HA sequences (dataset 2) does not provide a clear answer to the question, i.e., which model is the best. This may be caused by the noise in the original pairwise distance matrix, as well as the correlation between the rows of the matrix. The MDS method, however, provides an approximation to the proximity between sequences by representing them in a lower-dimensional space, while filtering out the noise in the pairwise distances. This motivated us to consider fitting Mclust on the location data obtained from MDS, instead of directly on the pairwise distance matrix. Figure 7 provides a snapshot of the 3D plot.

We used the 3D MDS to obtain the location data of influenza A (H7) HA sequences in a 3 dimensional space. By fitting the finite normal mixture model with the 3D coordinates of 1,168 sequences, we obtained a finite normal mixture with 10 clusters. Figure 7 shows a snapshot of the 10 clusters. The clustering result of the sequences is shown in Additional file 2: Table S2. The vast majority of sequences are assigned to a specific cluster with over 0.99 certainty, and only 50 sequences among 1,168 whose certainty is less than or equal to 0.99 (Table 2). We estimated the average substitution rate from the 1,168 sequences and used 0.10 as a threshold for the subset bootstrap. Table 3 reports the certainties of clusters from the subset bootstrap method and the standard bootstrap method, which shows the subset bootstrap method performs better than the standard bootstrap method. The certainties for cluster 6 and 8 are both below 0.50, indicating these 2 clusters are not well-supported by the bootstrapping data. We investigated the phylogenetic tree of the sequences in cluster 6 and found at least 2 subgroups in this cluster (Additional file 3: Figure S1). The low certainty values for some clusters might be contributed by the homogeneity variation of sequences within clusters.

**Table 2:** Certainties of influenza A (H7) HA sequences assigned to a specific cluster ^a^

Cluster ID	Strain Name	Certainty
1	A/chicken/NJ/17206/99	0.88
	A/Goose/New_Jersey/8600–3/98	0.97
2	A/chicken/FL/90348–4/01	0.54
	A/avian/NY/74211–2/00	0.98
	A/chicken/Pennsylvania/143586/2002	0.99
	A/avian/NY/81746–5/00	0.95
	A/avian/NY/70411–12/00	0.99
	A/unknown/NY/85161/2000	0.77
	A/chicken/NY/1398–6/99	0.97
	A/chicken/NY/22409–4/99	0.98
	A/avian/NY/76247–3/00	0.99
	A/Chicken/New_Jersey/20621/99	0.99
	A/Chicken/NJ/16224–6/99	0.99
3	A/mallard/Delaware/418/2005	0.96
6	A/turkey/England/647/77	0.84
8	A/swan/Shimane/42/1999	0.87
	A/turkey/Italy/4479/2004	0.73
	A/turkey/Italy/2856/2003	0.91
	A/turkey/Germany–NW/R655/2009	0.78
	A/turkey/Germany-NW/R655/2009	0.78
	A/duck/Mongolia/47/2012	0.76
	A/wild_goose/Dongting/PC0360/2012	0.80
	A/duck/Fukui/160104/2012	0.99
	A/duck/Iwate/0303001/2012	0.99
	A/mallard/Poland/01/08	0.82
	A/duck/Turkey/55/Cetinkaya/49/2006	0.90
	A/teal/Crimea/2027/2008	0.98
9	A/duck/Mongolia/720/2007	0.57
	A/turkey/Italy/3337/2004	0.96
	A/quail/Italy/3347/2004	0.96
	A/turkey/Italy/4130/2004	0.84
	A/turkey/Italy/3439/2004	0.89
	A/turkey/Italy/3829/2004	0.97
	A/turkey/Italy/3399/2004	0.82
	A/turkey/Italy/3477/2004	0.87
	A/turkey/Italy/3807/2004	0.87
	A/turkey/Italy/4042/2004	0.82
	A/turkey/Italy/2685/2003	0.59
	A/turkey/Italy/2043/2003	0.62
	A/duck/Italy/4609/2003	0.87
	A/quail/Italy/4610/2003	0.98
	A/chicken/Italy/1285/2000	0.98
	A/duck/Denmark/53–147–8/2008	0.90
	A/shoveler/Italy/2698–27/2006	0.85
	A/mallard/Netherlands/22/2007	0.65
	A/mallard/Sweden/95/2005	0.96
	A/Mallard/Sweden/S90597/2005	0.73
	A/chicken/England/4054/2006	0.96
	A/tufted_duck/PT/13771/2006	0.82
	A/mute_swan/Hungary/5973/2007	0.98

^a^ sequences not listed with a certainty of over 0.99

**Table 3:** The certainties of clusters and overall clustering for influenza A (H7) HA sequences

Cluster ID	1	2	3	4	5	6	7	8	9	10	Overall
Subset bootstrap (10 %)	0.84	0.85	0.93	0.73	0.67	0.43	0.66	0.40	0.90	0.98	0.82
Standard bootstrap method	0.72	0.78	0.83	0.39	0.65	0.34	0.59	0.24	0.78	0.90	0.67

## Conclusions

We formulated a clustering approach with the estimation of certainty and visualization of sequence data in 3D and applied it to analyse 2 datasets of influenza virus HA sequences. The results demonstrated the applicability of our approach in evolutionary clade assignment.

## Additional files

Additional file 1: Table S1. Cluster ID from Mclust, WHO designation of HPAI (H5N1) A HA sequences. Cluster ID from Mclust of the influenza A (H7) HA sequences. (DOCX 34 kb) Additional file 2: Table S2. Cluster ID from Mclust of the influenza A (H7) HA sequences. (DOCX 29 kb) Additional file 3: Figure S1. Phylogenetic tree of influenza A (H7) HA sequences in cluster 6. (PDF 2 kb)
