# Supplementary material for: Model-based clustering with certainty estimation: implication for clade assignment of influenza viruses
Source: BMC Bioinformatics. 2016 Jul 21;17:287. doi: 10.1186/s12859-016-1147-x (PMC4955158; doi:10.1186/s12859-016-1147-x)
Supplement: Additional file 2: Table S2. — Cluster ID from Mclust of the influenza A (H7) HA sequences. (DOCX 29 kb) [file 12859_2016_1147_MOESM2_ESM.docx]

Table S2. Cluster ID from Mclust of the influenza A (H7) HA sequences

| Cluster ID |  |
| --- | --- |
| 1 | A/cinnamon_teal/Bolivia/4537/2001 |
|  | A/chicken/Chile/176822/02 |
|  | A/turkey/Oregon/71 |
|  | A/turkey/Oregon/1971 |
|  | A/shorebird/Delaware_Bay/274/1994 |
|  | A/Rhea/North_Carolina/39482/93 |
|  | A/Rhea/North_Carolina/39482/93 |
|  | A/Quail/PA/20304/97 |
|  | A/mallard/Alberta/195/1989 |
|  | A/red_knot/NJ/325/1989 |
|  | A/turkey/Minnesota/1/1988 |
|  | A/ruddy_turnstone/DE/2378/1988 |
|  | A/green-winged_teal/ALB/228/1985 |
|  | A/mallard/Ohio/421/1987 |
|  | A/ruddy_turnstone/NJ/65/1985 |
|  | A/mallard_duck/Alberta/435/1985 |
|  | A/seal/Mass/1/80 |
|  | A/seal/Massachusetts/1/1980 |
|  | A/seal/Massachusetts/1/80 |
|  | A/turkey/Minnesota/1138/1980 |
|  | A/pheasant/Minnesota/917/1980 |
|  | A/turkey/MN/24552/1982 |
|  | A/turkey/Minnesota/1200/1980 |
|  | A/duck/Alberta/49/1976 |
|  | A/widgeon/Alberta/284/1977 |
|  | A/blue-winged_teal/ALB/295/1977 |
|  | A/mallard_duck/ALB/279/1977 |
|  | A/pintail/Alberta/21/1979 |
|  | A/mallard_duck/ALB/224/1977 |
|  | A/turkey/Tennessee/1/79 |
|  | A/shorebird/Delaware_Bay/53/2002 |
|  | A/ruddy_turnstone/DE/1538/00 |
|  | A/chicken/New_York/12273-11/1999 |
|  | A/laughing_gull/New_York/AI00-2455/2000 |
|  | A/mallard/Ohio/322/1998 |
|  | A/Chicken/NY/14714-2/1999 |
|  | A/chicken/New_York/14714-9/1999 |
|  | A/mallard/Minnesota/182761/1998 |
|  | A/red_knot/Delaware/650665/2002 |
|  | A/pintail/Minnesota/423/1999 |
|  | A/mallard/Maryland/423/2001 |
|  | A/American_black_duck/Maryland/424/2001 |
|  | A/mallard/MD/423/2001 |
|  | A/American_black_duck/Maryland/415/2001 |
|  | A/black_duck/MD/415/2001 |
|  | A/black_duck/Ohio/415/2001 |
|  | A/chicken/NJ/17206/99 |
|  | A/Goose/New_Jersey/8600-3/98 |
|  | A/Chicken/New_York/8030-2/96 |
|  | A/quail/NY/11430/99 |
|  | A/Quail/New_York/13989-51/98 |
|  | A/chicken/MD/MINH_MA/2004 |
|  | A/guineafowl/NJ/13246-9/98 |
|  | A/chicken/NY/3572/98 |
|  | A/chicken/NY/1387-8/98 |
|  | A/Turkey/Pennsylvania/7975/97 |
|  | A/Chicken/Pennsylvania/13552-1/98 |
|  | A/chicken/PA/19241/1997 |
|  | A/chicken/PA/149092-1/02 |
|  | A/Chicken/New_York/6777-3/97 |
|  | A/Chicken/Pennsylvania/11767-1/97 |
|  | A/duck/Guangdong/1/1996 |
|  | A/ruddy_turnstone/Delaware_Bay/135/1996 |
|  | A/ruddy_turnstone/Delaware_Bay/220/1995 |
|  | A/emu/TX/25414/1995 |
|  | A/turkey/Utah/24721-10/1995 |
|  | A/Turkey/New_York/4450-5/94 |
|  | A/duck/Alaska/3111/1993 |
|  | A/Chicken/New_York/13833-7/95 |
|  | A/chicken/NJ/15086-3/1994 |
|  | A/Chicken/New_York/13142-5/94 |
| 2 | A/environment/New_York/11678-1/2005 |
|  | A/chicken/New_York/11678-5/2005 |
|  | A/environment/New_York/11678-4/2005 |
|  | A/environment/New_York/11678-2/2005 |
|  | A/environment/New_York/11678-3/2005 |
|  | A/New_York/107/2003 |
|  | A/chicken/DE/VIVA/2004 |
|  | A/environment/New_York/30720-1/2005 |
|  | A/environment/New_York/30720-3/2005 |
|  | A/environment/New_York/31621-3/2005 |
|  | A/chicken/New_York/31621-6/2005 |
|  | A/environment/New_York/31621-1/2005 |
|  | A/environment/New_York/31621-5/2005 |
|  | A/guinea_fowl/New_York/31621-8/2005 |
|  | A/chicken/New_York/31621-9/2005 |
|  | A/environment/New_York/31621-2/2005 |
|  | A/chicken/New_York/87493-2/2005 |
|  | A/Muscovy_duck/New_York/87493-3/2005 |
|  | A/chicken/New_York/122501-1/2005 |
|  | A/turkey/New_York/122501-2/2005 |
|  | A/Muscovy_duck/New_York/19495-7/2006 |
|  | A/environment/New_York/143646-1/2005 |
|  | A/chicken/New_York/143646-2/2005 |
|  | A/Guinea_fowl/New_York/19495-6/2006 |
|  | A/environment/New_York/19495-1/2006 |
|  | A/chicken/New_York/19495-5/2006 |
|  | A/chicken/New_York/19495-3/2006 |
|  | A/chicken/New_York/19495-2/2006 |
|  | A/chicken/New_York/19495-4/2006 |
|  | A/duck/New_York/143646-5/2005 |
|  | A/pheasant/New_York/143646-4/2005 |
|  | A/guinea_fowl/New_York/143646-3/2005 |
|  | A/environment/New_York/3185-1/2006 |
|  | A/chicken/New_York/19499-1/2006 |
|  | A/chicken/New_York/19499/2005 |
|  | A/environment/NY/3185-28/2006 |
|  | A/environment/New_York/3185-4/2006 |
|  | A/environment/New_York/3185-2/2006 |
|  | A/environment/New_York/3185-5/2006 |
|  | A/environment/New_York/3185-3/2006 |
|  | A/chicken/New_York/46545-2/2006 |
|  | A/environment/New_York/46545-1/2006 |
|  | A/chicken/New_York/29047-4/2006 |
|  | A/environment/New_York/29047-2/2006 |
|  | A/environment/New_York/29047-1/2006 |
|  | A/environment/New_York/29047-3/2006 |
|  | A/chicken/DE/HOBO/2004 |
|  | A/guinea_fowl/New_York/22071/2005 |
|  | A/unknown/New_York/11646-5/2005 |
|  | A/Guinea_fowl/New_York/11646-3/2005 |
|  | A/chicken/New_York/11646-2/2005 |
|  | A/unknown/New_York/11646-6/2005 |
|  | A/unknown/New_York/11646-9/2005 |
|  | A/unknown/New_York/11646-8/2005 |
|  | A/Muscovy_duck/New_York/11646-4/2005 |
|  | A/chukar/New_York/11653-1/2005 |
|  | A/environment/New_York/81928-2/2005 |
|  | A/chicken/New_York/8391-2/2006 |
|  | A/guinea_fowl/New_York/8391-1/2006 |
|  | A/chicken/New_York/3181-5/2006 |
|  | A/environment/New_York/3181-4/2006 |
|  | A/environment/New_York/3181-1/2006 |
|  | A/chicken/New_York/3181-5/2006 |
|  | A/environment/New_York/19501-2/2006 |
|  | A/unknown/New_York/19501-5/2006 |
|  | A/guinea_fowl/New_York/19501-4/2006 |
|  | A/environment/New_York/19501-1/2006 |
|  | A/environment/New_York/3181-2/2006 |
|  | A/environment/New_York/19501-3/2006 |
|  | A/guinea_fowl/New_York/32084/2006 |
|  | A/chicken/New_York/10508/2005 |
|  | A/environment/New_York/139052/2005 |
|  | A/quail/New_York/63806-13/2005 |
|  | A/chicken/New_York/63806-11/2005 |
|  | A/chicken/New_York/63806-12/2005 |
|  | A/environment/New_York/63806-3/2005 |
|  | A/chicken/New_York/63806-8/2005 |
|  | A/environment/New_York/63806-4/2005 |
|  | A/chicken/New_York/16330/2005 |
|  | A/duck/New_York/88291-13/2005 |
|  | A/chicken/New_York/16326-3/2005 |
|  | A/chicken/New_York/16326-5/2005 |
|  | A/environment/New_York/88291-5/2005 |
|  | A/chicken/New_York/88291-6/2005 |
|  | A/chicken/New_York/88291-14/2005 |
|  | A/environment/New_York/88291-4/2005 |
|  | A/chicken/New_York/88291-15/2005 |
|  | A/duck/New_York/88291-12/2005 |
|  | A/environment/New_York/88291-2/2005 |
|  | A/environment/New_York/88291-1/2005 |
|  | A/environment/New_York/88291-3/2005 |
|  | A/unknown/New_York/88291-16/2005 |
|  | A/chicken/New_York/88291-10/2005 |
|  | A/Guinea_fowl/New_York/88291-9/2005 |
|  | A/chicken/New_York/88291-8/2005 |
|  | A/unknown/New_York/88291-17/2005 |
|  | A/chicken/New_York/88291-11/2005 |
|  | A/chicken/New_York/16326-4/2005 |
|  | A/environment/New_York/16326-2/2005 |
|  | A/environment/New_York/16326-1/2005 |
|  | A/environment/New_York/23164-1/2005 |
|  | A/chicken/New_York/23164-7/2005 |
|  | A/muscovy_duck/New_York/23164-10/2005 |
|  | A/chicken/New_York/23164-4/2005 |
|  | A/chicken/New_York/23164-9/2005 |
|  | A/chukar/New_York/23164-13/2005 |
|  | A/environment/New_York/23164-2/2005 |
|  | A/guinea_fowl/New_York/23164-3-05/2005 |
|  | A/chicken/New_York/23164-5/2005 |
|  | A/turkey/New_York/23164-11/2005 |
|  | A/chicken/New_York/23164-6/2005 |
|  | A/chicken/NJ/294508-12/2004 |
|  | A/environment/New_York/98616-2/2005 |
|  | A/chicken/New_York/42217/2005 |
|  | A/guineafowl/NY/4649-18/2006 |
|  | A/duck/New_York/98616-5/2005 |
|  | A/duck/New_York/98616-4/2005 |
|  | A/Guinea_fowl/New_York/101276-1/2005 |
|  | A/unknown/New_York/11675/2005 |
|  | A/environment/New_York/22067-4/2005 |
|  | A/environment/New_York/22067-2/2005 |
|  | A/duck/New_York/22067-11/2005 |
|  | A/guinea_fowl/New_York/22067-10/2005 |
|  | A/chicken/New_York/22067-9/2005 |
|  | A/chicken/New_York/22067-8/2005 |
|  | A/environment/New_York/22067-6/2005 |
|  | A/chicken/New_York/22067-12/2005 |
|  | A/chicken/New_York/22067-7/2005 |
|  | A/environment/New_York/22067-5/2005 |
|  | A/environment/New_York/22067-3/2005 |
|  | A/environment/New_York/22067-1/2005 |
|  | A/chicken/New_York/79672/2005 |
|  | A/environment/New_York/26792-4/2005 |
|  | A/environment/New_York/26792-2/2005 |
|  | A/environment/New_York/26792-1/2005 |
|  | A/environment/New_York/26792-3/2005 |
|  | A/unknown/New_York/98616-6/2006 |
|  | A/Guinea_fowl/New_York/98616-3/2005 |
|  | A/chicken/New_York/37982-4/2005 |
|  | A/environment/New_York/37982-2/2005 |
|  | A/guinea_fowl/New_York/37982-3/2005 |
|  | A/environment/New_York/37982-1/2005 |
|  | A/duck/New_York/21211-6/2005 |
|  | A/chicken/New_York/21211-1/2005 |
|  | A/unknown/New_York/13479-3/2005 |
|  | A/unknown/New_York/13479-4/2005 |
|  | A/unknown/New_York/13479-2/2005 |
|  | A/unknown/New_York/13479-5/2005 |
|  | A/chicken/New_York/10196-5/2005 |
|  | A/chicken/New_York/10196-4/2005 |
|  | A/chicken/New_York/21211-2/2005 |
|  | A/unknown/New_York/13479-1/2005 |
|  | A/chukar/New_York/21211-7/2005 |
|  | A/avian/NY/118353-1/2001 |
|  | A/chicken/NJ/118878-5/01 |
|  | A/guinea_fowl/NJ/119063-8/2001 |
|  | A/guineafowl/MA/148081-11/02 |
|  | A/chicken/NJ/151244-18/02 |
|  | A/chicken/NJ/150383-7/02 |
|  | A/chicken/FL/90348-4/01 |
|  | A/chicken/CT/260413-2/2003 |
|  | A/chicken/New_York/23165-9/2005 |
|  | A/chicken/New_York/23165-11/2005 |
|  | A/muscovy_duck/New_York/23165-13/2005 |
|  | A/guinea_fowl/New_York/23165-12/2005 |
|  | A/chicken/New_York/23165-7/2005 |
|  | A/chicken/New_York/23165-10/2005 |
|  | A/environment/New_York/23165-4/2005 |
|  | A/environment/New_York/23165-5/2005 |
|  | A/chicken/New_York/23165-6/2005 |
|  | A/environment/New_York/23165-1/2005 |
|  | A/quail/New_York/89641/2005 |
|  | A/chicken/New_York/30732-8/2005 |
|  | A/chicken/New_York/30732-11/2005 |
|  | A/chicken/New_York/30732-10/2005 |
|  | A/chicken/New_York/30732-9/2005 |
|  | A/environment/New_York/30732-4/2005 |
|  | A/chicken/New_York/30732-6/2005 |
|  | A/environment/New_York/30732-1/2005 |
|  | A/muscovy_duck/New_York/30732-13/2005 |
|  | A/quail/New_York/30732-12/2005 |
|  | A/turkey/New_York/30732-7/2005 |
|  | A/environment/New_York/30732-3/2005 |
|  | A/environment/New_York/30732-2/2005 |
|  | A/avian/NY/73063-6/00 |
|  | A/environment/NY/260422-10/2003 |
|  | A/chicken/NY/119256-7/01 |
|  | A/avian/NY/74211-2/00 |
|  | A/chicken/NY/30749-3/00 |
|  | A/environmental/NY/268925-10/2003 |
|  | A/environment/NY/241365-18/2003 |
|  | A/turkey/VA/67/02 |
|  | A/chicken/NJ/608/02 |
|  | A/chicken/VA/32/02 |
|  | A/Turkey/NC/11165/02 |
|  | A/turkey/VA/66/02 |
|  | A/chicken/NY/119055-7/2001 |
|  | A/chicken/NY/119055-7/01 |
|  | A/turkey/VA/55/02 |
|  | A/avian/NY/7729-6/00 |
|  | A/unknown/NY/_117932-11/2001 |
|  | A/turkey/VA/158512/2002 |
|  | A/chicken/Pennsylvania/143586/2002 |
|  | A/avian/NY/81746-5/00 |
|  | A/avian/NY/70411-12/00 |
|  | A/unknown/NY/85161/2000 |
|  | A/chicken/NY/1398-6/99 |
|  | A/chicken/NY/34173-3/99 |
|  | A/chicken/NY/22409-4/99 |
|  | A/unknown/NY/120485-5/2001 |
|  | A/avian/NY/76247-3/00 |
|  | A/chicken/NJ/15814-9/99 |
|  | A/Chicken/New_Jersey/20621/99 |
|  | A/chicken/NY/21586-8/99 |
|  | A/Chicken/NJ/16224-6/99 |
|  | A/Chicken/NY/14858-12/99 |
| 3 | A/ruddy_turnstone/New_Jersey/622/2006 |
|  | A/ruddy_turnstone/New_Jersey/604/2006 |
|  | A/ruddy_turnstone/New_Jersey/518/2006 |
|  | A/ruddy_turnstone/New_Jersey/490/2006 |
|  | A/sanderling/New_Jersey/369/2006 |
|  | A/ruddy_turnstone/New_Jersey/549/2006 |
|  | A/ruddy_turnstone/New_Jersey/215/2006 |
|  | A/ruddy_turnstone/Delaware/880/2006 |
|  | A/ruddy_turnstone/Delaware/752/2006 |
|  | A/laughing_gull/Delaware_Bay/42/2006 |
|  | A/laughing_gull/Delaware_Bay/46/2006 |
|  | A/laughing_gull/Delaware/42/06 |
|  | A/sanderling/New_Jersey/355/2006 |
|  | A/ruddy_turnstone/New_Jersey/612/2006 |
|  | A/ruddy_turnstone/New_Jersey/175/2006 |
|  | A/ruddy_turnstone/New_Jersey/594/2006 |
|  | A/ruddy_turnstone/New_Jersey/527/2006 |
|  | A/ruddy_turnstone/New_Jersey/200/2006 |
|  | A/ruddy_turnstone/New_Jersey/176/2006 |
|  | A/ruddy_turnstone/Delaware/891/2006 |
|  | A/ruddy_turnstone/Delaware/887/2006 |
|  | A/ruddy_turnstone/New_Jersey/204/2006 |
|  | A/ruddy_turnstone/New_Jersey/563/2006 |
|  | A/laughing_gull/Delaware_Bay/6/2006 |
|  | A/shorebird/Delaware_Bay/332/2006 |
|  | A/shorebird/Delaware_Bay/332/2006 |
|  | A/shorebird/Delaware_Bay/513/2006 |
|  | A/ruddy_turnstone/New_Jersey/576/2006 |
|  | A/ruddy_turnstone/New_Jersey/562/2006 |
|  | A/ruddy_turnstone/Delaware/789/2006 |
|  | A/ruddy_turnstone/Delaware/779/2006 |
|  | A/ruddy_turnstone/Delaware/769/2006 |
|  | A/sanderling/New_Jersey/125/2006 |
|  | A/red_knot/New_Jersey/96/2006 |
|  | A/ruddy_turnstone/New_Jersey/171/2006 |
|  | A/ruddy_turnstone/New_Jersey/199/2006 |
|  | A/ruddy_turnstone/New_Jersey/189/2006 |
|  | A/ruddy_turnstone/New_Jersey/183/2006 |
|  | A/ruddy_turnstone/New_Jersey/166/2006 |
|  | A/ruddy_turnstone/Delaware/760/2006 |
|  | A/red_knot/New_Jersey/253/2006 |
|  | A/shorebird/Delaware_Bay/560/2006 |
|  | A/shorebird/Delaware/22/06 |
|  | A/sanderling/New_Jersey/AI06-125/2006 |
|  | A/red_knot/New_Jersey/AI06-096/2006 |
|  | A/ruddy_turnstone/New_Jersey/224/2006 |
|  | A/sanderling/New_Jersey/148/2006 |
|  | A/ruddy_turnstone/New_Jersey/577/2006 |
|  | A/ruddy_turnstone/Delaware_Bay/281/2006 |
|  | A/ruddy_turnstone/Delaware_Bay/282/2006 |
|  | A/shorebird/Delaware_Bay/552/2006 |
|  | A/ruddy_turnstone/New_Jersey/196/2006 |
|  | A/ruddy_turnstone/Delaware_Bay/279/2006 |
|  | A/ruddy_turnstone/Delaware_Bay/283/2006 |
|  | A/ruddy_turnstone/New_Jersey/589/2006 |
|  | A/ruddy_turnstone/New_Jersey/565/2006 |
|  | A/ruddy_turnstone/New_Jersey/195/2006 |
|  | A/ruddy_turnstone/Delaware/893/2006 |
|  | A/ruddy_turnstone/DE/892/2006 |
|  | A/ruddy_turnstone/New_Jersey/569/2006 |
|  | A/ruddy_turnstone/New_Jersey/564/2006 |
|  | A/ruddy_turnstone/Delaware/778/2006 |
|  | A/ruddy_turnstone/Delaware/AI06-778/2006 |
|  | A/ruddy_turnstone/New_Jersey/AI06-179/2006 |
|  | A/cinnamon_teal/Mexico/2817/2006 |
|  | A/northern_shoveler/Washington/44249-752/2006 |
|  | A/northern_shoveler/Washington/44249-749/2006 |
|  | A/northern_shoveler/Washington/44249-783/2006 |
|  | A/black_scoter/New_Brunswick/00003/2009 |
|  | A/black_scoter/New_Brunswick/00010/2009 |
|  | A/black_scoter/New_Brunswick/00014/2009 |
|  | A/northern_pintail/Interior_Alaska/8MP0262R2/2008 |
|  | A/mallard/Ohio/11OS2010/2011 |
|  | A/mallard/Ohio/11OS2033/2011 |
|  | A/mallard/Wisconsin/10OS3171/2010 |
|  | A/northern_shoveler/Wisconsin/10OS3226/2010 |
|  | A/mallard/California/1390/2010 |
|  | A/mallard/Interior_Alaska/10BM07575R0/2010 |
|  | A/northern_pintail/Illinois/10OS3959/2010 |
|  | A/northern_pintail/Interior_Alaska/10BM02539R0/2010 |
|  | A/mallard/Interior_Alaska/10CH00016R0/2010 |
|  | A/northern_pintail/Interior_Alaska/10BM09015R0/2010 |
|  | A/mallard/Interior_Alaska/10BM04564R1/2010 |
|  | A/northern_pintail/Interior_Alaska/10BM11038R0/2010 |
|  | A/northern_pintail/Interior_Alaska/10BM11366R0/2010 |
|  | A/northern_pintail/Interior_Alaska/10BM09481R0/2010 |
|  | A/mallard/Interior_Alaska/10BM09539R0/2010 |
|  | A/mallard/Interior_Alaska/10BM09528R0/2010 |
|  | A/mallard/Interior_Alaska/10BM09548R0/2010 |
|  | A/northern_pintail/Interior_Alaska/10BM06303R0/2010 |
|  | A/mallard/Interior_Alaska/10BM10829R0/2010 |
|  | A/northern_pintail/Interior_Alaska/10BM11945R0/2010 |
|  | A/mallard/Interior_Alaska/10BM10876R0/2010 |
|  | A/mallard/Interior_Alaska/10BM10861R0/2010 |
|  | A/mallard/Interior_Alaska/10BM10859R0/2010 |
|  | A/northern_pintail/Interior_Alaska/10BM10853R0/2010 |
|  | A/northern_pintail/Interior_Alaska/10BM10790R0/2010 |
|  | A/mallard/Interior_Alaska/10BM10787R0/2010 |
|  | A/northern_pintail/Interior_Alaska/10BM10906R0/2010 |
|  | A/northern_pintail/Interior_Alaska/10BM10476R0/2010 |
|  | A/northern_pintail/Interior_Alaska/10BM10166R0/2010 |
|  | A/mallard/Interior_Alaska/10BM09563R0/2010 |
|  | A/mallard/Interior_Alaska/10BM11434R0/2010 |
|  | A/mallard/Interior_Alaska/10BM10811R0/2010 |
|  | A/northern_pintail/Interior_Alaska/10BM10809R0/2010 |
|  | A/northern_pintail/Interior_Alaska/10BM10807R0/2010 |
|  | A/northern_pintail/Interior_Alaska/10BM10805R0/2010 |
|  | A/northern_pintail/Interior_Alaska/10BM10801R0/2010 |
|  | A/mallard/Interior_Alaska/10BM10324R0/2010 |
|  | A/northern_pintail/Interior_Alaska/10BM09832R0/2010 |
|  | A/mallard/Interior_Alaska/10BM09818R0/2010 |
|  | A/northern_pintail/Interior_Alaska/10BM11208R0/2010 |
|  | A/mallard/Interior_Alaska/10BM08884R0/2010 |
|  | A/mallard/Interior_Alaska/10BM08883R0/2010 |
|  | A/mallard/Interior_Alaska/10BM08874R0/2010 |
|  | A/northern_pintail/Interior_Alaska/10BM08400R0/2010 |
|  | A/northern_pintail/Interior_Alaska/10BM09348R0/2010 |
|  | A/northern_pintail/Interior_Alaska/10BM09347R0/2010 |
|  | A/northern_pintail/Interior_Alaska/10BM08586R0/2010 |
|  | A/mallard/Interior_Alaska/10BM05860R0/2010 |
|  | A/mallard/Interior_Alaska/10BM05347R0/2010 |
|  | A/northern_pintail/Interior_Alaska/10BM07120R0/2010 |
|  | A/northern_pintail/Interior_Alaska/10BM07399R0/2010 |
|  | A/northern_pintail/Interior_Alaska/10BM07121R0/2010 |
|  | A/northern_pintail/Interior_Alaska/10BM07029R0/2010 |
|  | A/northern_pintail/Interior_Alaska/10BM06720R0/2010 |
|  | A/mallard/Interior_Alaska/10BM06838R0/2010 |
|  | A/northern_pintail/Interior_Alaska/10BM06306R0/2010 |
|  | A/northern_pintail/Interior_Alaska/10BM07737R0/2010 |
|  | A/northern_pintail/Interior_Alaska/10BM07458R0/2010 |
|  | A/northern_pintail/Interior_Alaska/10BM07106R0/2010 |
|  | A/northern_pintail/Interior_Alaska/10BM08705R0/2010 |
|  | A/northern_pintail/Interior_Alaska/10BM07469R0/2010 |
|  | A/mallard/Interior_Alaska/10BM12534R0/2010 |
|  | A/mallard/Interior_Alaska/10BM08120R0/2010 |
|  | A/mallard/Interior_Alaska/10BM07085R0/2010 |
|  | A/mallard/Interior_Alaska/10BM07072R0/2010 |
|  | A/northern_pintail/Interior_Alaska/10BM09181R0/2010 |
|  | A/mallard/Interior_Alaska/10BM08100R0/2010 |
|  | A/mallard/Interior_Alaska/10BM07501R0/2010 |
|  | A/northern_pintail/Interior_Alaska/10BM07114R0/2010 |
|  | A/mallard/Interior_Alaska/10BM07092R0/2010 |
|  | A/mallard/Interior_Alaska/10BM07066R0/2010 |
|  | A/mallard/Interior_Alaska/10BM06911R0/2010 |
|  | A/mallard/Interior_Alaska/10BM06909R0/2010 |
|  | A/mallard/Interior_Alaska/10BM06905R0/2010 |
|  | A/northern_pintail/Interior_Alaska/10BM06899R0/2010 |
|  | A/northern_pintail/Interior_Alaska/10BM06895R0/2010 |
|  | A/northern_pintail/Interior_Alaska/10BM06524R0/2010 |
|  | A/northern_shoveler/Mississippi/11OS289/2011 |
|  | A/gadwall/Missouri/10OS4731/2010 |
|  | A/guinea_fowl/Nebraska/17096-1/2011 |
|  | A/goose/Nebraska/17097-4/2011 |
|  | A/American_green-winged_teal/Illinois/10OS4014/2010 |
|  | A/American_green-winged_teal/Illinois/10OS3368/2010 |
|  | A/American_green-winged_teal/Illinois/10OS3329/2010 |
|  | A/northern_shoveler/Mississippi/11OS202/2011 |
|  | A/northern_shoveler/Missouri/10OS4632/2010 |
|  | A/American_green-winged_teal/Mississippi/11OS255/2011 |
|  | A/American_green-winged_teal/Mississippi/11OS250/2011 |
|  | A/American_black_duck/Wisconsin/10OS3949/2010 |
|  | A/northern_shoverl/Mississippi/11OS145/2011 |
|  | A/northern_shoveler/Missouri/10OS4750/2010 |
|  | A/mallard/Missouri/10MO0551/2010 |
|  | A/mallard/Missouri/220/2009 |
|  | A/mallard/Missouri/10MO053/2010 |
|  | A/green-winged_teal/New_Brunswick/00392/2010 |
|  | A/mallard/Nova_Scotia/00372/2010 |
|  | A/American_black_duck/New_Brunswick/00344/2010 |
|  | A/common_goldeneye/Wisconsin/10OS4202/2010 |
|  | A/blue-winged_Teal/North_Dakota/AI09-3760/2009 |
|  | A/northern_shoveler/Mississippi/09OS643/2009 |
|  | A/American_green-winged_teal/Mississippi/09OS046/2009 |
|  | A/environment/Maryland/1637/2006 |
|  | A/environment/Maryland/1631/2006 |
|  | A/environment/Maryland/267/2006 |
|  | A/environment/Maryland/1626/2006 |
|  | A/environment/Maryland/276/2006 |
|  | A/environment/Maryland/261/2006 |
|  | A/northern_shoveler/California/JN1447/2007 |
|  | A/environment/Indiana/08OS2885/2008 |
|  | A/mallard/Wisconsin/08OS2844/2008 |
|  | A/ruddy_turnstone/Delaware_Bay/124/2007 |
|  | A/ruddy_turnstone/Delaware_Bay/123/2007 |
|  | A/ruddy_turnstone/Delaware_Bay/121/2007 |
|  | A/ruddy_turnstone/Delaware_Bay/108/2007 |
|  | A/mallard/California/HKWF1971/2007 |
|  | A/green_winged_teal/California/AKS1370/2008 |
|  | A/northern_shoveler/California/HKWF2031/2008 |
|  | A/mallard/Nova_Scotia/02286/2007 |
|  | A/American_black_duck/New_Brunswick/04388/2007 |
|  | A/American_black_duck/NB/2538/2007 |
|  | A/American_black_duck/New_Brunswick/02490/2007 |
|  | A/American_black_duck/New_Brunswick/02493/2007 |
|  | A/Canada_goose/BC/3752/2007 |
|  | A/northern_shoveler/California/44287-364/2007 |
|  | A/northern_shoveler/California/28327/2007 |
|  | A/cinnamon_teal/California/JN611/2006 |
|  | A/American_green-winged_teal/California/44287-713/2007 |
|  | A/American_green-winged_teal/California/28855/2007 |
|  | A/American_green-winged_teal/California/44242-906/2007 |
|  | A/northern_shoveler/California/44287-164/2007 |
|  | A/American_green-winged_teal/California/44287-084/2007 |
|  | A/northern_shoveler/California/27820/2007 |
|  | A/Northern_shoveler/NC/6412-052/2005 |
|  | A/northern_shoveler/North_Carolina/674-516/2005 |
|  | A/blue-winged_teal/Ohio/566/2006 |
|  | A/northern_shoveler/North_Carolina/6412-050/2005 |
|  | A/mallard/Delaware/418/2005 |
|  | A/American_green-winged_teal/California/44287-305/2007 |
|  | A/American_green-winged_teal/California/28228/2007 |
|  | A/northern_shoveler/California/44287-179/2007 |
|  | A/northern_shoveler/California/27985/2007 |
|  | A/duck/AB/AFLBs8734c16/2007 |
|  | A/northern_shoveler/California/HKWF1372C/2007 |
|  | A/northern_shoveler/Washington/44249-664/2006 |
|  | A/environment/California/7451/2010 |
|  | A/green-winged_teal/California/11275/2008 |
|  | A/green-winged_teal/California/1841/2009 |
|  | A/northern_shoveler/California/HKWF1026/2007 |
|  | A/blue-winged_teal/Guatemala/CIP049-02/2008 |
|  | A/blue-winged_teal/Guatemala/CIP049-01/2008 |
| 4 | A/green-winged_teal/Ohio/648/2004 |
|  | A/green-winged_teal/MD/648/2004 |
|  | A/blue-winged_teal/Ohio/658/2004 |
|  | A/blue-winged_teal/MD/658/2004 |
|  | A/blue-winged_teal/Ohio/658/2004 |
|  | A/chicken/British_Columbia/NS-01827-4/2004 |
|  | A/chicken/Canada/314514-1/2005 |
|  | A/blue-winged_teal/Texas/578588/2002 |
|  | A/blue-winged_teal/Texas/578575/2002 |
|  | A/Duck/PA/143585/01 |
|  | A/blue-winged_teal/Texas/578585/2002 |
|  | A/mallard/Alberta/24/01 |
|  | A/mallard/Alberta/34/2001 |
|  | A/mallard/Alberta/24/01 |
|  | A/mallard/Alberta/22/2001 |
|  | A/mallard/Interior_Alaska/6MP0984/2006 |
|  | A/cinnamon_teal/California/JN1310/2007 |
|  | A/mallard/Alberta/243/2006 |
|  | A/mallard/Alberta/243/2006 |
| 5 | A/duck/Victoria/512/2007 |
|  | A/duck/Tasmania/277/2007 |
|  | A/duck/Victoria/1976 |
|  | A/chicken/Victoria/1976 |
|  | A/chicken/Victoria/1/1985 |
|  | A/chicken/Victoria/224/1992 |
|  | A/chicken/Victoria/1/92 |
|  | A/chicken/Queensland/1994 |
|  | A/chicken/Queensland/667/95 |
|  | A/emu/New_South_Wales/775/1997 |
|  | A/chicken/NSW/1/1997 |
|  | A/chicken/New_South_Wales/327/1997 |
|  | A/chicken/New_South_Wales/2/1997 |
|  | A/duck/Taiwan/Ya103/1993 |
|  | A/duck/Taiwan/33/1993 |
|  | A/chicken/Rostock/45/1934 |
|  | A/chicken/Germany/1934 |
|  | A/ts1/1/A/FPV/Rostock/1934 |
|  | A/FPV/Rostock/1934 |
|  | A/FPV/Dutch/27 |
|  | A/fowl/Weybridge/1933 |
|  | A/fowl/Dobson/1927 |
|  | A/FPV/Dutch/1927 |
|  | A/chicken/Brescia/1902 |
| 6 | A/mallard/New_Zealand/1365-355/2005 |
|  | A/quail/Aichi/4/2009 |
|  | A/quail/Aichi/1/2009 |
|  | A/quail/Aichi/6/2009 |
|  | A/quail/Aichi/5/2009 |
|  | A/quail/Aichi/3/2009 |
|  | A/quail/Aichi/2/2009 |
|  | A/duck/Vietnam/NCVD-197/2009 |
|  | A/duck/Hubei/126/1985 |
|  | A/duck/Hongkong/301/72 |
|  | A/duck/Hong_Kong/301/1978 |
|  | A/duck/Hong_Kong/293/78 |
|  | A/duck/Hong_Kong/293/1978 |
|  | A/turkey/England/1963 |
|  | A/chicken/Pakistan/34668/1995 |
|  | A/duck/Taiwan/2009 |
|  | A/parrot/Northern_Ireland/VF-73-67/73 |
|  | A/chicken/Chakwal/NARC-35/2001 |
|  | A/macaw/England/626/80 |
|  | A/goose/Leipzig/187/7/1979 |
|  | A/chicken/Leipzig/79 |
|  | A/chicken/Germany/01/1979 |
|  | A/goose/Leipzig/192/7/1979 |
|  | A/goose/Leipzig/137/8/1979 |
|  | A/turkey/England/647/77 |
|  | A/tern/Potsdam/343/1979 |
|  | A/duck/Potsdam/15/1980 |
|  | A/turkey/England/192-328/79 |
| 7 | A/chicken/Pakistan/1/2004 |
|  | A/chicken/Karachi/131/2004 |
|  | A/chicken/Pakistan/3/2004 |
|  | A/chicken/Pakistan/2/2004 |
|  | A/chicken/Karachi/132/2004 |
|  | A/chicken/Karachi/133/2004 |
|  | A/chicken/Pakistan/447/95 |
|  | A/chicken/Pakistan/CR2/95 |
|  | A/chicken/Karachi/NARC-23/2003 |
|  | A/chicken/Chakwal/NARC-46/2003 |
|  | A/chicken/Chakwal/NARC-46/2003 |
|  | A/chicken/Karachi/NARC-23/2003 |
|  | A/chicken/Rawalpindi/NARC68/2002 |
|  | A/chicken/Murree/NARC-1/1995 |
|  | A/chicken/Murree/NARC-01/1995 |
|  | A/chicken/Karachi/NARC-100/2004 |
|  | A/chicken/Karachi/NARC-100/2004 |
|  | A/chicken/Karachi/135/2004 |
|  | A/chicken/Karachi/134/2004 |
|  | A/chicken/Chakwal/NARC-148/2004 |
|  | A/chicken/Chakwal/NARC-148/2004 |
|  | A/chicken/Karachi/146/2004 |
|  | A/chicken/Karachi/147/2004 |
|  | A/chicken/Pakistan/c1998/1998 |
|  | A/duck/Fukui/1/2004 |
|  | A/duck/Hokkaido/143/03 |
|  | A/duck/Taiwan/4201/99 |
|  | A/wild_bird_feces/Hadoree/8/2003 |
|  | A/wild_bird_feces/Korea/HDR16/2003 |
|  | A/duck/Mongolia/867/2002 |
|  | A/duck/Hokkaido/Vac-2/04 |
|  | A/duck/Thailand/CU-LM7288C/2010 |
|  | A/duck/Thailand/CU-LM7306C/2010 |
|  | A/duck/Thailand/CU-LM7288T/2010 |
|  | A/duck/Thailand/CU-LM7302T/2010 |
|  | A/duck/Thailand/CU-LM7308C/2010 |
|  | A/duck/Thailand/CU-LM7308T/2010 |
|  | A/duck/Thailand/CU-LM7283T/2010 |
|  | A/duck/Thailand/CU-LM7297T/2010 |
|  | A/duck/Thailand/CU-LM7306T/2010 |
|  | A/duck/Thailand/CU-LM7283C/2010 |
|  | A/duck/Thailand/CU-LM7294T/2010 |
|  | A/duck/Thailand/CU-LM7285C/2010 |
|  | A/duck/Thailand/CU-LM7298T/2010 |
|  | A/duck/Thailand/CU-LM7279T/2010 |
|  | A/duck/Thailand/CU-LM7280C/2010 |
|  | A/duck/Thailand/CU-LM7291C/2010 |
|  | A/duck/Thailand/CU-LM7284C/2010 |
|  | A/duck/Thailand/CU-LM7301T/2010 |
|  | A/duck/Thailand/CU-LM7291T/2010 |
|  | A/duck/Korea/BC10/2007 |
|  | A/northern_pintail/Aomori/1001/2008 |
|  | A/northern_pintail/Aomori/372/2008 |
|  | A/northern_pintail/Miyagi/674/2008 |
|  | A/duck/Tsukuba/922/2008 |
|  | A/duck/Tsukuba/30/2007 |
|  | A/mallard/Korea/GG2/2007 |
|  | A/northern_pintail/Akita/1370/2008 |
|  | A/northern_pintail/Akita/1369/2008 |
|  | A/northern_pintail/Akita/1367/2008 |
|  | A/northern_pintail/Akita/1368/2008 |
|  | A/duck/Mongolia/147/2008 |
|  | A/duck/Mongolia/119/2008 |
|  | A/duck/Mongolia/128/2008 |
|  | A/duck/Korea/A117/10 |
|  | A/duck/Korea/JSM/10 |
|  | A/duck/Korea/LSY/10 |
|  | A/duck/Shimane/137/2006 |
|  | A/duck/Shimane/83/2006 |
|  | A/wild_bird/Korea/A330/09 |
|  | A/wild_bird/Korea/A331/09 |
|  | A/duck/Hokkaido/1/2010 |
|  | A/duck/Chiba/20/2009 |
|  | A/duck/Korea/A76/10 |
|  | A/duck/Korea/A79/10 |
|  | A/duck/Tsukuba/700/2007 |
|  | A/duck/Tsukuba/664/2007 |
|  | A/duck/Shiga/B149/2007 |
|  | A/duck/Shimane/18/2006 |
|  | A/Shanghai/1/2013 |
|  | A/chicken/Zhejiang/DTID-ZJU01/2013 |
|  | A/Taiwan/1/2013 |
|  | A/Chicken/Hangzhou/50/2013 |
|  | A/Shanghai/4664T/2013 |
|  | A/Hangzhou/2/2013 |
|  | A/Nanjing/1/2013 |
|  | A/Zhejiang/HZ1/2013 |
|  | A/Hangzhou/3/2013 |
|  | A/chicken/Jiangsu/K27/2013 |
|  | A/chicken/Jiangsu/K27/2013 |
|  | A/environment/Nanjing/2913/2013 |
|  | A/Chicken/Shanghai/S1053/2013 |
|  | A/Zhejiang/1/2013 |
|  | A/Zhejiang/01/2013 |
|  | A/Hangzhou/1/2013 |
|  | A/Environment/Hangzhou/37/2013 |
|  | A/Environment/Hangzhou/34/2013 |
|  | A/Nanchang/1/2013 |
|  | A/Chicken/Hangzhou/48/2013 |
|  | A/Pigeon/Shanghai/S1069/2013 |
|  | A/Shanghai/4/2013 |
|  | A/Shanghai/3/2013 |
|  | A/Environment/Shanghai/S1088/2013 |
|  | A/Fujian/1/2013 |
|  | A/Environment/Hangzhou/109/2013 |
|  | A/Zhejiang/2/2013 |
|  | A/chicken/Jiangsu/K89/2013 |
|  | A/Zhejiang/DTID-ZJU01/2013 |
|  | A/Anhui/1/2013 |
|  | A/Shanghai/2/2013 |
|  | A/duck/Zhejiang/DK16/2013 |
|  | A/duck/Zhejiang/DK10/2013 |
|  | A/duck/Zhejiang/10/2011 |
|  | A/duck/Zhejiang/12/2011 |
|  | A/duck/Zhejiang/11/2011 |
|  | A/duck/Zhejiang/2/2011 |
|  | A/duck/Heinersdorf/S495/6/86 |
|  | A/chicken/Jena/1816/87 |
|  | A/gull/Italy/692-2/93 |
|  | A/psittacine/Italy/1/91 |
|  | A/turkey/Israel/Ramon/79 |
|  | A/Mallard/Sweden/56/02 |
|  | A/non-psittacine/England-Q/1985/89 |
|  | A/softbill/California/33445-136/1992 |
|  | A/softbill/CA/33445-158/1992 |
|  | A/ostrich/South_Africa/M320/96 |
|  | A/turkey/Ireland/PV74/1995 |
|  | A/England/AV877/1996 |
|  | A/England/268/1996 |
|  | A/turkey/Northern_Ireland/VF-1545_C5/98 |
|  | A/turkey/Ireland/PV8/1995 |
|  | A/ostrich/South_Africa/1069/91 |
|  | A/ostrich/South_Africa/1991 |
|  | A/ostrich/Zimbabwe/222/96 |
|  | A/duck/Nanchang/1904/1992 |
|  | A/common_iora_/Singapore/F89/95 |
|  | A/fairy_bluebird/Singapore/F92/94 |
|  | A/conure/England/766/94 |
|  | A/Pekin_robin/California/30412/1994 |
|  | A/parakeet/Netherlands/267497/94 |
|  | A/parrot/England/1174/94 |
| 8 | A/chicken/Netherlands/12014794/2012 |
|  | A/chicken/Neth/11008327/11 |
|  | A/Branta_canadensis/Belgium/13000-9-2/2010 |
|  | A/chicken/Netherlands/11004875/11 |
|  | A/swan/Czech_Republic/5416/2011 |
|  | A/chicken/Germany-NI/R874/2010 |
|  | A/swan/Shimane/42/1999 |
|  | A/turkey/Italy/4479/2004 |
|  | A/turkey/Italy/2856/2003 |
|  | A/turkey/Germany-NW/R655/2009 |
|  | A/turkey/Germany-NW/R655/2009 |
|  | A/duck/Mongolia/47/2012 |
|  | A/duck/Thailand/CU-10531T/2011 |
|  | A/duck/Thailand/CU-10507T/2011 |
|  | A/duck/Thailand/CU-10524C/2011 |
|  | A/duck/Thailand/CU-10530T/2011 |
|  | A/duck/Thailand/CU-10512C/2011 |
|  | A/duck/Thailand/CU-10530C/2011 |
|  | A/duck/Thailand/CU-10534C/2011 |
|  | A/duck/Thailand/CU-10518C/2011 |
|  | A/duck/Thailand/CU-10525T/2011 |
|  | A/wild_goose/Dongting/PC0360/2012 |
|  | A/duck/Fukui/160104/2012 |
|  | A/duck/Fukui/161101/2011 |
|  | A/duck/Gunma/466/2011 |
|  | A/duck/Gunma/11-610-118/2012 |
|  | A/duck/Hokkaido/W63/2011 |
|  | A/duck/Hokkaido/W62/2011 |
|  | A/duck/Thailand/CU-9744C_/2010 |
|  | A/duck/Thailand/CU-9754C/2010 |
|  | A/duck/Iwate/0303001/2012 |
|  | A/duck/Iwate/301007/2012 |
|  | A/duck/Iwate/301012/2012 |
|  | A/duck/Chiba/25-51-14/2013 |
|  | A/duck/Tochigi/090206/2013 |
|  | A/Baer's_pochard/HuNan/414/2010 |
|  | A/duck/Mongolia/129/2010 |
|  | A/mallard/Italy/3397-65/2008 |
|  | A/swan/Slovenia/53/2009 |
|  | A/goose/Czech_Republic/1848-T14/2009 |
|  | A/goose/Czech_Republic/1848/2009 |
|  | A/mallard/Poland/01/08 |
|  | A/duck/Turkey/55/Cetinkaya/49/2006 |
|  | A/teal/Crimea/2027/2008 |
|  | A/Anas_crecca/Spain/1460/2008 |
|  | A/Anas_crecca/Spain/1460/2008 |
|  | A/guinea_fowl/Italy/407/2008 |
|  | A/mallard/Italy/794-18/2008 |
|  | A/teal/Italy/794-3/2008 |
| 9 | A/mallard/Sweden/7206/2004 |
|  | A/mallard/Germany/NVP41/2004 |
|  | A/mallard/Sweden/105/2002 |
|  | A/Mallard/Sweden/105/02 |
|  | A/duck/Mongolia/720/2007 |
|  | A/Mallard/Sweden/S90735/2003 |
|  | A/Mallard/Sweden/87/02 |
|  | A/Mallard/Sweden/104/02 |
|  | A/Mallard/Sweden/102/02 |
|  | A/Mallard/Sweden/103/02 |
|  | A/Mallard/Sweden/94/02 |
|  | A/Mallard/Sweden/91/02 |
|  | A/Mallard/Sweden/93/02 |
|  | A/Mallard/Sweden/100/02 |
|  | A/Mallard/Sweden/107/02 |
|  | A/Mallard/Sweden/106/02 |
|  | A/Mallard/Sweden/92/02 |
|  | A/Mallard/Sweden/82/02 |
|  | A/mallard/Sweden/85/2002 |
|  | A/Mallard/Sweden/85/02 |
|  | A/turkey/Italy/3337/2004 |
|  | A/quail/Italy/3347/2004 |
|  | A/turkey/Italy/4130/2004 |
|  | A/turkey/Italy/3439/2004 |
|  | A/turkey/Italy/3829/2004 |
|  | A/turkey/Italy/3399/2004 |
|  | A/turkey/Italy/3477/2004 |
|  | A/turkey/Italy/3807/2004 |
|  | A/turkey/Italy/4042/2004 |
|  | A/chicken/Italy/4616/2003 |
|  | A/turkey/Italy/2962/2003 |
|  | A/turkey/Italy/2987/2003 |
|  | A/turkey/Italy/3620/2003 |
|  | A/mallard/Italy/199/01 |
|  | A/mallard/Italy/33/01 |
|  | A/mallard/Italy/43/2001 |
|  | A/chicken/Italy/2240/2003 |
|  | A/Guinea_fowl/Italy/266184/02 |
|  | A/guinea_fowl/Italy/1613/2003 |
|  | A/turkey/Italy/4036/2003 |
|  | A/turkey/Italy/4608/2003 |
|  | A/turkey/Italy/251/2003 |
|  | A/turkey/Italy/9737/2002 |
|  | A/turkey/Italy/9739/2002 |
|  | A/turkey/Italy/1010/2003 |
|  | A/chicken/Italy/8093/2002 |
|  | A/turkey/Italy/8534/2002 |
|  | A/turkey/Italy/8000/2002 |
|  | A/chicken/Italy/270638/02 |
|  | A/turkey/Italy/8535/02 |
|  | A/turkey/Italy/220158/2002 |
|  | A/turkey/Italy/214845/02 |
|  | A/turkey/Italy/220158/2002 |
|  | A/turkey/Italy/214845/2002 |
|  | A/turkey/Italy/8307/2002 |
|  | A/chicken/Italy/682/2003 |
|  | A/chicken/Italy/682/2003 |
|  | A/turkey/Italy/9369/2002 |
|  | A/turkey/Italy/8458/2002 |
|  | A/turkey/Italy/8912/2002 |
|  | A/turkey/Italy/9374/2002 |
|  | A/turkey/Italy/9102/2002 |
|  | A/turkey/Italy/8834/2002 |
|  | A/turkey/Italy/8651/2002 |
|  | A/turkey/Italy/9742/2002 |
|  | A/turkey/Italy/9314/2002 |
|  | A/turkey/Italy/2685/2003 |
|  | A/turkey/Italy/2043/2003 |
|  | A/duck/Italy/4609/2003 |
|  | A/quail/Italy/4610/2003 |
|  | A/duck/Mongolia/47/01 |
|  | A/turkey/Germany/R11/2001 |
|  | A/mallard/Netherlands/12/2000 |
|  | A/mallard/Netherlands/12/00 |
|  | A/mallard/Netherlands/12/00 |
|  | A/chicken/Italy/322/2001 |
|  | A/turkey/Italy/3283/1999 |
|  | A/turkey/Italy/2732/1999 |
|  | A/turkey/Italy/2732/1999 |
|  | A/turkey/Italy/2715/99 |
|  | A/turkey/Italy/3489/99 |
|  | A/turkey/Italy/3488/1999 |
|  | A/turkey/Italy/3185/99 |
|  | A/turkey/Italy/4301/1999 |
|  | A/turkey/Italy/4295/1999 |
|  | A/turkey/Italy/3675/1999 |
|  | A/turkey/Italy/3675/99 |
|  | A/chicken/Italy/1067/1999 |
|  | A/turkey/Italy/3889/99 |
|  | A/chicken/Italy/1391/1999 |
|  | A/chicken/Italy/1082/1999 |
|  | A/chicken/Italy/1067/99 |
|  | A/turkey/Italy/1265/99 |
|  | A/turkey/Italy/4603/1999 |
|  | A/turkey/Italy/4602/99 |
|  | A/chicken/Italy/4575/99 |
|  | A/turkey/Italy/4169/99 |
|  | A/turkey/Italy/4169/99 |
|  | A/turkey/Italy/3775/99 |
|  | A/turkey/Italy/4073/99 |
|  | A/turkey/Italy/2379/2000 |
|  | A/turkey/Italy/977/1999 |
|  | A/turkey/Italy/977/99 |
|  | A/turkey/Italy/2984/2000 |
|  | A/quail/Italy/396/2000 |
|  | A/chicken/Italy/1285/2000 |
|  | A/ostrich/Italy/2332/00 |
|  | A/ostrich/Italy/2332/00 |
|  | A/pekin_duck/Italy/1848/2000 |
|  | A/turkey/Italy/4580/99 |
|  | A/turkey/Italy/4708/1999 |
|  | A/turkey/Italy/12598/99 |
|  | A/Turkey/Italy/13489/99 |
|  | A/Chicken/Italy/267/00 |
|  | A/chicken/Italy/445/99 |
|  | A/Italy/445/99 |
|  | A/chicken/Italy/445/99 |
|  | A/turkey/Italy/1084/2000 |
|  | A/chicken/Italy/2335/2000 |
|  | A/guinea_fowl/Italy/155/2000 |
|  | A/chicken/Italy/445/1999 |
|  | A/quail/Italy/4992/1999 |
|  | A/duck/Italy/551/2000 |
|  | A/chicken/Italy/13474/99 |
|  | A/Chicken/Italy/13307/99 |
|  | A/Turkey/Italy/13467/99 |
|  | A/Turkey/Italy/13468/99 |
|  | A/Turkey/Italy/335/00 |
|  | A/ostrich/Italy/984/00 |
|  | A/ostrich/Italy/984/00 |
|  | A/chicken/Italy/5093/99 |
|  | A/chicken/Italy/5093/99 |
|  | A/chicken/Italy/4789/1999 |
|  | A/chicken/Italy/4746/1999 |
|  | A/ostrich/Italy/1038/2000 |
|  | A/turkey/Italy/4426/1999 |
|  | A/turkey/Italy/4426/2000 |
|  | A/turkey/Italy/3560/99 |
|  | A/turkey/Italy/4482/1999 |
|  | A/turkey/Italy/4294/99 |
|  | A/turkey/Italy/4644/99 |
|  | A/turkey/Italy/4482/1999 |
|  | A/duck/Denmark/53-147-8/2008 |
|  | A/shoveler/Italy/2698-27/2006 |
|  | A/mallard/Netherlands/22/2007 |
|  | A/mallard/Netherlands/29/2006 |
|  | A/mallard/Netherlands/33/2006 |
|  | A/mallard/Sweden/95/2005 |
|  | A/Mallard/Sweden/S90597/2005 |
|  | A/mallard/Netherlands/9/2005 |
|  | A/chicken/England/4054/2006 |
|  | A/chicken/England/4266/2006 |
|  | A/tufted_duck/PT/13771/2006 |
|  | A/mute_swan/Hungary/5973/2007 |
| 10 | A/chicken/Germany/R28/03 |
|  | A/chicken/Netherlands/03009975/03 |
|  | A/chicken/Netherlands/03010703/03 |
|  | A/chicken/Netherlands/03009190/03 |
|  | A/chicken/Netherlands/03014339/03 |
|  | A/chicken/Netherlands/03009337/03 |
|  | A/chicken/Netherlands/03011728/03 |
|  | A/chicken/Netherlands/03009196/03 |
|  | A/chicken/Netherlands/03009885/03 |
|  | A/chicken/Netherlands/03010040/03 |
|  | A/chicken/Netherlands/03009888/03 |
|  | A/chicken/Netherlands/03009858/03 |
|  | A/chicken/Netherlands/03009833/03 |
|  | A/chicken/Netherlands/03009122/03 |
|  | A/chicken/Netherlands/03009754/03 |
|  | A/chicken/Netherlands/03010736/03 |
|  | A/chicken/Netherlands/03009334/03 |
|  | A/chicken/Netherlands/03009923/03 |
|  | A/chicken/Netherlands/03009886/03 |
|  | A/chicken/Netherlands/03009884/03 |
|  | A/chicken/Netherlands/03009863/03 |
|  | A/chicken/Netherlands/03009857/03 |
|  | A/chicken/Netherlands/03009828/03 |
|  | A/chicken/Netherlands/03009787/03 |
|  | A/chicken/Netherlands/03010735/03 |
|  | A/chicken/Netherlands/03009862/03 |
|  | A/chicken/Netherlands/03009432/03 |
|  | A/chicken/Netherlands/03009755/03 |
|  | A/chicken/Netherlands/03009276/03 |
|  | A/chicken/Netherlands/03009281/03 |
|  | A/chicken/Netherlands/03009145/03 |
|  | A/chicken/Netherlands/03009119/03 |
|  | A/chicken/Netherlands/03009048/03 |
|  | A/chicken/Netherlands/03008327/03 |
|  | A/chicken/Netherlands/03009859/03 |
|  | A/chicken/Netherlands/03009830/03 |
|  | A/chicken/Netherlands/03009403/03 |
|  | A/chicken/Netherlands/03009189/03 |
|  | A/chicken/Netherlands/03009059/03 |
|  | A/chicken/Netherlands/03009083/03 |
|  | A/chicken/Netherlands/03008082/03 |
|  | A/chicken/Netherlands/03007158/03 |
|  | A/chicken/Netherlands/03010734/03 |
|  | A/chicken/Netherlands/03010539/03 |
|  | A/chicken/Netherlands/03010039/03 |
|  | A/chicken/Netherlands/03009860/03 |
|  | A/chicken/Netherlands/03009338/03 |
|  | A/chicken/Netherlands/03009171/03 |
|  | A/chicken/Netherlands/03009144/03 |
|  | A/chicken/Netherlands/03009012/03 |
|  | A/chicken/Netherlands/03008929/03 |
|  | A/chicken/Netherlands/03003445/03 |
|  | A/chicken/Netherlands/03008379/03 |
|  | A/chicken/Netherlands/03012221/03 |
|  | A/chicken/belgium/06383/2003 |
|  | A/turkey/Netherlands/03010821/03 |
|  | A/chicken/Netherlands/03013011/03 |
|  | A/chicken/Netherlands/03013094/03 |
|  | A/chicken/Netherlands/03008885/03 |
|  | A/chicken/Netherlands/03009892/03 |
|  | A/chicken/Netherlands/03013457/03 |
|  | A/chicken/Netherlands/03013456/03 |
|  | A/chicken/Netherlands/03013104/03 |
|  | A/chicken/Netherlands/03003109/03 |
|  | A/chicken/Netherlands/03003245/03 |
|  | A/chicken/Netherlands/03008200/03 |
|  | A/chicken/Netherlands/03008344/03 |
|  | A/chicken/Netherlands/03005432/03 |
|  | A/chicken/Netherlands/03009938/03 |
|  | A/chicken/Netherlands/03008374/03 |
|  | A/chicken/Netherlands/03009127/03 |
|  | A/chicken/Netherlands/03008199/03 |
|  | A/chicken/Netherlands/03008110/03 |
|  | A/chicken/Netherlands/03008233/03 |
|  | A/chicken/Netherlands/03008489/03 |
|  | A/chicken/Netherlands/03008926/03 |
|  | A/chicken/Netherlands/03009430/03 |
|  | A/chicken/Netherlands/03009430/03 |
|  | A/chicken/Netherlands/03009656/03 |
|  | A/turkey/Netherlands/03009333/03 |
|  | A/chicken/Netherlands/03008128/03 |
|  | A/chicken/Netherlands/03009791/03 |
|  | A/chicken/Netherlands/03009126/03 |
|  | A/chicken/Netherlands/03009123/03 |
|  | A/chicken/Netherlands/03009104/03 |
|  | A/chicken/Netherlands/03008451/03 |
|  | A/chicken/Netherlands/03009274/03 |
|  | A/chicken/Netherlands/03008377/03 |
|  | A/chicken/Netherlands/03008113/03 |
|  | A/chicken/Netherlands/03008060/03 |
|  | A/chicken/Netherlands/03003531/03 |
|  | A/chicken/Netherlands/03009890/03 |
|  | A/chicken/Netherlands/03009120/03 |
|  | A/chicken/Netherlands/03009889/03 |
|  | A/chicken/Netherlands/03009921/03 |
|  | A/chicken/belgium/06175/2003 |
|  | A/chicken/Netherlands/03012010/03 |
|  | A/chicken/Netherlands/03012785/03 |
|  | A/chicken/Netherlands/03009008/03 |
|  | A/turkey/Netherlands/03011419/03 |
|  | A/turkey/Netherlands/03010598/03 |
|  | A/chicken/Netherlands/03008379/03 |
|  | A/chicken/Netherlands/03009398/03 |
|  | A/chicken/Netherlands/03004209/03 |
|  | A/chicken/Netherlands/03003242/03 |
|  | A/chicken/Netherlands/03012009/03 |
|  | A/chicken/Netherlands/03011422/03 |
|  | A/chicken/Netherlands/03013833/03 |
|  | A/chicken/Netherlands/03012012/03 |
|  | A/chicken/Netherlands/03008331/03 |
|  | A/chicken/Netherlands/03009080/03 |
|  | A/chicken/Netherlands/03008381/03 |
|  | A/chicken/Netherlands/03002974/03 |
|  | A/chicken/Netherlands/03012011/03 |
|  | A/chicken/Netherlands/03011061/03 |
|  | A/turkey/Netherlands/03010737/03 |
|  | A/turkey/Netherlands/03010495/03 |
|  | A/chicken/Netherlands/03009831/03 |
|  | A/chicken/Netherlands/03003441/03 |
|  | A/chicken/Netherlands/03008059/03 |
|  | A/chicken/Netherlands/03003325/03 |
|  | A/chicken/Netherlands/03003403/03 |
|  | A/chicken/Netherlands/03002994/03 |
|  | A/chicken/Netherlands/03013531/03 |
|  | A/chicken/Netherlands/03013010/03 |
|  | A/turkey/Netherlands/03012387/03 |
|  | A/chicken/Netherlands/03012013/03 |
|  | A/chicken/Netherlands/03011766/03 |
|  | A/chicken/Netherlands/UN242/03 |
|  | A/chicken/Netherlands/03011236/03 |
|  | A/chicken/Netherlands/03012915/03 |
|  | A/chicken/Netherlands/03009939/03 |
|  | A/chicken/Netherlands/03007917/03 |
|  | A/turkey/Netherlands/03010350/03 |
|  | A/chicken/Netherlands/03010351/03 |
|  | A/chicken/Netherlands/03009940/03 |
|  | A/chicken/Netherlands/03009431/03 |
|  | A/chicken/Netherlands/03008932/03 |
|  | A/chicken/Netherlands/03010134/03 |
|  | A/chicken/Netherlands/03008235/03 |
|  | A/chicken/Netherlands/03009789/03 |
|  | A/chicken/Netherlands/03003572/03 |
|  | A/chicken/Netherlands/03009942/03 |
|  | A/turkey/Netherlands/03009788/03 |
|  | A/chicken/Netherlands/03009192/03 |
|  | A/chicken/Netherlands/03008375/03 |
|  | A/chicken/Netherlands/03008383/03 |
|  | A/chicken/Netherlands/03012386/03 |
|  | A/chicken/Netherlands/03009304/03 |
|  | A/chicken/Netherlands/03003257/03 |
|  | A/chicken/Netherlands/621629/03 |
|  | A/turkey/Netherlands/03007922/03 |
|  | A/chicken/Netherlands/03003402/03 |
|  | A/chicken/Netherlands/03009146/03 |
|  | A/turkey/Netherlands/03008130/03 |
|  | A/chicken/Netherlands/03005418/03 |
|  | A/chicken/Netherlands/03012365/03 |
|  | A/Netherlands/219/03 |
|  | A/chicken/Netherlands/03010132/03 |
|  | A/chicken/Netherlands/03010132/03 |
|  | A/chicken/Netherlands/03013566/03 |
|  | A/chicken/belgium/06775/2003 |
|  | A/chicken/belgium/06600/2003 |
|  | A/chicken/Netherlands/03013633/03 |
|  | A/chicken/Netherlands/03011063/03 |
|  | A/chicken/Netherlands/03012872/03 |
|  | A/chicken/Netherlands/03005333/03 |
|  | A/chicken/Netherlands/03005334/03 |
|  | A/chicken/Netherlands/03006730/03 |
|  | A/chicken/Netherlands/03006724/03 |
|  | A/chicken/Netherlands/03003113/03 |
|  | A/chicken/Netherlands/03003106/03 |
|  | A/turkey/Netherlands/03008232/03 |
|  | A/chicken/Netherlands/03003633/03 |
|  | A/chicken/Netherlands/621572/03 |
|  | A/chicken/Netherlands/03003244/03 |
|  | A/chicken/Netherlands/03004210/03 |
|  | A/chicken/Netherlands/03003256/03 |
|  | A/chicken/Netherlands/03006728/03 |
|  | A/chicken/Netherlands/03006725/03 |
|  | A/chicken/Netherlands/03002971/03 |
|  | A/chicken/Netherlands/03003010/03 |
|  | A/chicken/Netherlands/03008084/03 |
|  | A/chicken/Netherlands/03008034/03 |
|  | A/chicken/Netherlands/03007894/03 |
|  | A/chicken/Netherlands/03008998/03 |
|  | A/chicken/Netherlands/03008271/03 |
|  | A/chicken/Netherlands/03003111/03 |
|  | A/chicken/Netherlands/03006936/03 |
|  | A/turkey/Netherlands/03003567/03 |
|  | A/chicken/Netherlands/03003110/03 |
|  | A/chicken/Netherlands/03009694/03 |
|  | A/chicken/Netherlands/03009941/03 |
|  | A/chicken/Netherlands/03013101/03 |
|  | A/chicken/Netherlands/03013079/03 |
|  | A/chicken/Netherlands/03012969/03 |
|  | A/chicken/Netherlands/03009007/03 |
|  | A/turkey/Netherlands/03007999/03 |
|  | A/chicken/Netherlands/03011884/03 |
|  | A/chicken/Netherlands/03009124/03 |
|  | A/chicken/Netherlands/03009044/03 |
|  | A/chicken/Netherlands/03008201/03 |
|  | A/chicken/Netherlands/03007782/03 |
|  | A/chicken/Netherlands/03003258/03 |
|  | A/chicken/Netherlands/621576/03 |
|  | A/chicken/Netherlands/03003214/03 |
|  | A/chicken/Netherlands/03003214/03 |
|  | A/chicken/Netherlands/03002973/03 |
|  | A/domestic_duck/Netherlands/621627/03 |
|  | A/turkey/Netherlands/03010496/03 |
|  | A/chicken/Netherlands/03009125/03 |
|  | A/chicken/Netherlands/03003415/03 |
|  | A/chicken/Netherlands/03009014/03 |
|  | A/chicken/Netherlands/03008514/03 |
|  | A/chicken/Netherlands/03008450/03 |
|  | A/chicken/Netherlands/621571/03 |
|  | A/chicken/Netherlands/03005431/03 |
|  | A/turkey/Netherlands/03003568/03 |
|  | A/chicken/Netherlands/03003401/03 |
|  | A/chicken/Netherlands/03003246/03 |
|  | A/chicken/Netherlands/03003255/03 |
|  | A/chicken/Netherlands/03003370/03 |
|  | A/chicken/Netherlands/03003243/03 |
|  | A/chicken/Netherlands/03003105/03 |
|  | A/chicken/Netherlands/03003096/03 |
|  | A/chicken/Netherlands/03003008/03 |
|  | A/chicken/Netherlands/03003217/03 |
|  | A/chicken/Netherlands/03003212/03 |
|  | A/chicken/Netherlands/621560/03 |
|  | A/chicken/Netherlands/03003093/03 |
|  | A/chicken/Netherlands/03013102/03 |
|  | A/chicken/Netherlands/03012874/03 |
|  | A/chicken/Netherlands/03012135/03 |
|  | A/chicken/Netherlands/03005336/03 |
|  | A/chicken/Netherlands/03005499/03 |
|  | A/chicken/Netherlands/03008000/03 |
|  | A/chicken/Netherlands/03007960/03 |
|  | A/chicken/Netherlands/03003092/03 |
|  | A/chicken/Netherlands/03003340/03 |
|  | A/chicken/Netherlands/621612/03 |
|  | A/chicken/Netherlands/621559/03 |
|  | A/chicken/Netherlands/2586/2003 |
|  | A/chicken/Netherlands/1/03 |
|  | A/Netherlands/33/03 |
