# Supplementary figures and images for: Model-based clustering with certainty estimation: implication for clade assignment of influenza viruses
Source: BMC Bioinformatics. 2016 Jul 21;17:287. doi: 10.1186/s12859-016-1147-x (PMC4955158; doi:10.1186/s12859-016-1147-x)

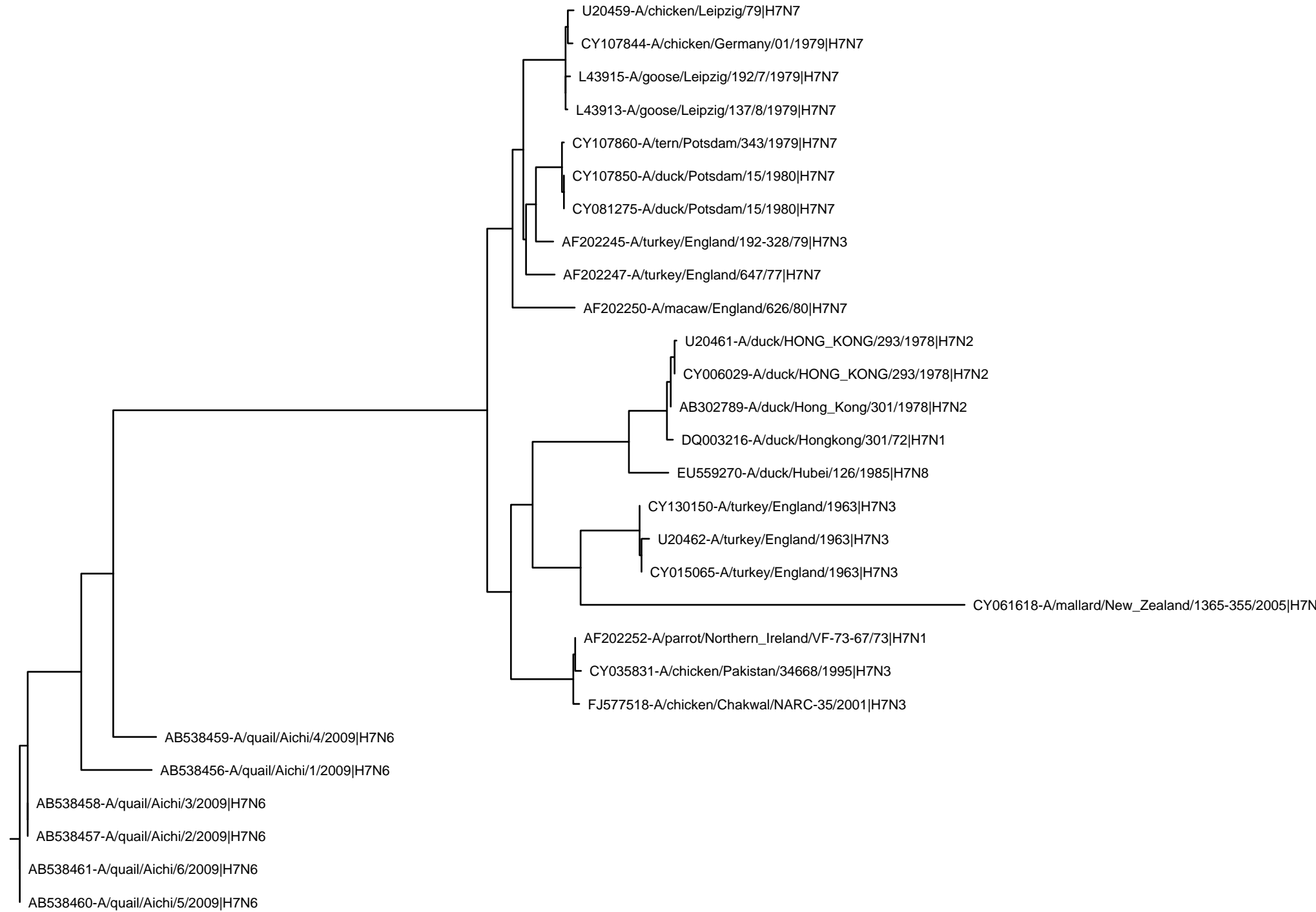

0.03

Supplement: Additional file 3: Figure S1. — Phylogenetic tree of influenza A (H7) HA sequences in cluster 6. (PDF 2 kb) [file 12859_2016_1147_MOESM3_ESM.pdf]
